# Bacterial Biofilm Growth on 3D-Printed Materials

**DOI:** 10.3389/fmicb.2021.646303

**Published:** 2021-05-28

**Authors:** Donald C. Hall, Phillip Palmer, Hai-Feng Ji, Garth D. Ehrlich, Jarosław E. Król

**Affiliations:** ^1^Department of Chemistry, Drexel University, Philadelphia, PA, United States; ^2^Center for Advanced Microbial Processing and Center for Surgical Infections and Biofilms, Institute for Molecular Medicine and Infectious Disease, Department of Microbiology and Immunology, Philadelphia, PA, United States; ^3^Department of Otolaryngology-Head and Neck Surgery, Drexel University College of Medicine, Philadelphia, PA, United States

**Keywords:** biofilm, antimicrobial properties, 3D printing, bacterial infections, 3D structures, surface topology, PLA polymer

## Abstract

Recent advances in 3D printing have led to a rise in the use of 3D printed materials in prosthetics and external medical devices. These devices, while inexpensive, have not been adequately studied for their ability to resist biofouling and biofilm buildup. Bacterial biofilms are a major cause of biofouling in the medical field and, therefore, hospital-acquired, and medical device infections. These surface-attached bacteria are highly recalcitrant to conventional antimicrobial agents and result in chronic infections. During the COVID-19 pandemic, the U.S. Food and Drug Administration and medical officials have considered 3D printed medical devices as alternatives to conventional devices, due to manufacturing shortages. This abundant use of 3D printed devices in the medical fields warrants studies to assess the ability of different microorganisms to attach and colonize to such surfaces. In this study, we describe methods to determine bacterial biofouling and biofilm formation on 3D printed materials. We explored the biofilm-forming ability of multiple opportunistic pathogens commonly found on the human body including *Escherichia coli*, *Pseudomonas aeruginosa*, and *Staphylococcus aureus* to colonize eight commonly used polylactic acid (PLA) polymers. Biofilm quantification, surface topography, digital optical microscopy, and 3D projections were employed to better understand the bacterial attachment to 3D printed surfaces. We found that biofilm formation depends on surface structure, hydrophobicity, and that there was a wide range of antimicrobial properties among the tested polymers. We compared our tested materials with commercially available antimicrobial PLA polymers.

## Introduction

Biofouling is the process of microorganisms attaching to solid inanimate surfaces as biofilms. It is estimated that biofouling costs billions of dollars per year and is a problem within many fields of science and industry, including the medical fields (Vladkova, 2009; Gule et al., 2016). One of the most recent areas impacted by biofouling is 3D-printed materials. 3D printed materials have become common in medical settings, especially in the area of prosthetic limbs (Poologasundarampillai and Nommeots-Nomm, 2017; Paul et al., 2018; Sun, 2018). Such 3D-printed prosthetics have not yet been studied for their ability to reduce biofilm attachments nor their overall antimicrobial properties. This contrasts with conventional prosthetic limbs, which have been studied and developed to have antibiofilm and antimicrobial properties (Nablo et al., 2001).

The major cause of biofouling in the medical setting is bacterial cell attachment (Bixler Gregory and Bhushan, 2012; Harding and Reynolds, 2014; Damodaran and Murthy, 2016; Zander and Becker, 2018). Biofilms can be considered as a multicellular phase of growth in the life cycle of bacteria (Donlan, 2002; Sauer et al., 2002; Kostakioti et al., 2013). Once cells first attach to a surface, they extrude a matrix and develop cooperative behaviors resulting in the development of multicellular biofilm form. Often biofilms are metabolically resistant to antibiotics and antimicrobials, sometimes up to 1,000 × their planktonic counterparts (Hall-Stoodley et al., 2008; Wolcott and Ehrlich, 2008; Ito et al., 2009) due to triggering of the stringent response (Hall et al., 2020) and other gene expression changes (Costerton et al., 2007; Lewis, 2008; Wood et al., 2013). Bacterial biofilms are also the hot spots for horizontal gene transfer events, especially multidrug resistance plasmid conjugation (Król et al., 2011; Król et al., 2013). This makes the eradication of biofilms difficult in a medical setting as they can only be removed by physical means. These facts make it necessary to understand all aspects of medical devices being used in these medical settings. 3D printing has been utilized in casts, prosthetics, food products, and containers. All these applications have the potential for allowing biofilm development due to their proximity to bacteria and food sources.

Use of 3D-printed prosthetics is increasing because of their relative cost-effectiveness compared to that of conventional prosthetic devices (Zuniga et al., 2015; Choonara et al., 2016). While the average conventional prostatic can cost upwards of U.S. $50,000, a 3D-printed prosthetic can be made for less than U.S. $1000 (TASOM Engineers, 2018). This significant reduction in cost is due to the ability to quickly adapt models for the user’s needs (Manero et al., 2019). 3D-printed models can be easily switched out when parts are damaged and as children grow.

Because 3D-printed materials are being used more frequently as medical materials, there is an overwhelming need to develop antifouling 3D-printing materials. There are many materials already on the market that utilize the simple method of impregnating the polymer filament with nanoparticles, fibers, or metal flakes (Zuniga, 2018). However, methods of studying and understanding biofilm formation and antimicrobial properties of 3D-printed materials are lacking.

The resolution on 3D printers is improving rapidly and high-resolution printers are affordable even as desktop devices. The resolution at which a typical extruder printer operates is 200 μm between layers and all polymers lead to an inherent surface roughness upon printing. These rough surfaces can provide an ideal environment for the initial attachment of bacteria and subsequent formation of biofilms (Mitik-Dineva et al., 2009).

In this study, we worked with several common polylactic acid (PLA) polymers doped with various materials and printed using a common extruder 3D printer. We report several polymers that have antifouling properties and develop a model for studying biofilm formation on these materials. We compare surface properties, roughness, and hydrophobicity in regard to the attachment and amount of biofilms. We also report several novel techniques to map and understand how biofilms attach to 3D printed materials. Notably, we developed surface analysis of biofilms using 3D optical profilometry to study the biofilm points of attachment and to gain understanding of how the surface roughness of 3D-printed materials contributes to biofilm attachment.

## Materials and Methods

PLA polymers (Table 1) were used with a MakerBot Replicator2 3D printer (MarkerBot, New York, NY, United States) (standard settings: 0.2 mm, 15% infill) to print standard (3 × 1 inch) slides, rings (outer diameter, 15 mm; inner diameter, 10 mm; height, 15.25 mm), and pins (head diameter, 10 mm; pin diameter, 5.5 mm; height, 15-17 mm) designed by a SketchUP Pro2018 (Supplementary Figure 1). Plastic (Rinzl) and glass microscope slides (Fisherbrand) were purchased from Amazon and Fisher Scientific, respectively. All STL files are available upon request.

**TABLE 1:** 3D polymers used in this study.

**Material**	**Manufacturer**	**Composition**	**Abbreviation**
Brass 3D Printer Filament	Yoyi 3D	33- 40% metal powder and 67-60% PLA	BRS
Copper 3D Printer Filament	Yoyi 3D	33% metal powder and 67% PLA	CU
Aluminum 3D Printer Filament	Yoyi 3D	33% metal powder and 67% PLA	AL
3D Printer Filament Frosted Bronze	AMOLEN	20% metal powder and 80% PLA	BRZ
Carbon Fiber Black	3D Solutech	70% PLA and 30% carbon fiber.	CF
3D PLA Wood color	AmazonBasic	70% polymer and 30% wood.	WD
Black Soft PLA	MatterHackers		SoftPLA
Silver Metal 3D Printer PLA Filament	3D Solutech	Silver Dye (no metal infill)	PLAS
**Antimicrobial polymers**			
Purement	Purement BnK Chemical Company	Copper	Pur
PLActive	Copper3D	Copper	PLAc
Antibacterial PLA	XYZ Printing	Silver ions	XYZ

### Surface Characterization

#### Surface Free Energy – Hydrophobicity

The Dataphysics (Charlotte, NC, United States) Optical Contact Angle OCA 15EC measuring system was used with MilliQ^®^ water to measure surface wettability. The surface angle of 1 μL drop was recorded in 4 different locations along the 3D printed slides and controls.

### Surface Topography – Contact and Optical Profilometers

A Mitutoyo (Kanagawa, Japan) JS210 contact profilometer was used to analyze surface properties of the materials using standard parameters like JIS, VDA, ISO-1997 and ANSI. Standard linear (2D) parameters (Ra, Rq, Rz, Rp, Rv, Rsk, Rku, Rc, RSm, RDq, Rmr, Rdc, Rt, Rk, Rpk, Rvk) (Supplementary Table 1) were examined and the averages from 4 horizontal and vertical reads were calculated.

A Leica (Leica United States) DVM6 digital microscope and MountainsMap^®^ ver.7.4 (Digital Surf SARL, France) were used to collect and analyze 3D images. Standard surface 3D parameters S (Sq, Ssk, Sku, Sp, Sv, Sz, Sa, Sz, Smr, Smc, Sxp, Sdc) (Supplementary Table 2) and 2D parameters R (Rp, Rv, Rz, Rc, Rt, Ra, Rq, Rdc, Rsk, Rku, Rmr) were measured and averages were calculated based on at least 3 pictures and 3 profiles.

### Bacterial Growth - Viability Assay

Printed out 3D rings were sterilized for 30 min in 70% ethanol followed by washing twice in sterile water or autoclaving in MilliQ^®^ water (15 min) or just a dry cycle (15 min- sterilization/10min-drying). Rings were placed in 24-well plates (Supplementary Figure 1B). Eight hundred microliters of 100-fold diluted overnight (18 h) cultures of *Escherichia coli C, Pseudomonas aeruginosa* PA01, and *Staphylococcus aureus* ATCC 25923 in LB Miller medium were aliquoted into the wells. Plates were placed in Tecan (Tecan Inc., United States) Infinite 200Pro plate reader executing 15 min shaking and OD_600_ read cycles at 37°C for 18 h.

### Bacterial Adhesion

Overnight cultures (25 mL) of bacterial strains in LB Miller medium were transferred into 50-mL conical Falcon tubes (Supplementary Figure 2). Slides were sterilized with 75% ethanol for 30 min following by washing twice in sterile water. Slides were incubated with bacterial cultures for 2 h at 37°C and shaken at 50 rpm. Planktonic cells were washed out by submerging the slides 4 times in 50 mL phosphate-buffered saline (PBS). Biofilm bacteria were detached from the surface by vortexing 3 min at 1,000 rpm (Mini-G, SPEX-Sample-Prep) in 25 mL PBS. Serial dilutions were plated by a drop-titer method on LB agar plates. Colonies (CFU) were counted after overnight incubation at 37°C.

### Biofilm Assay

Printed pins were sterilized (autoclaved 15 min/10 min) and placed into 500 μL of 100x dilutions of bacterial overnight cultures in 48-well plates (Corning United States). Biofilms were grown for 3 days at 37°C (50 rpm) in Forma Model 3,950 incubator (> 90% humidity). Pins were submerged in the culture entire time and no change in liquid level was observed. After 3 days pins were removed and bacterial cell densities (OD_600_) were measured in the medium. Pins were washed twice in PBS and placed into 700 μL BacTiter-Glo^TM^ Reagent (Promega) (Herten et al., 2017) for 15 min. Two hundred microliters were transferred into 96-well white plates and the luminescence was measured using a Tecan Infinite 200Pro plate reader (integration time 1,000 s). Pins submerged in the sterile media were used as a control and these values were subtracted from the biofilm luminescence. Luminescence of the reagent was used as the control. Experiments were run in triplicate.

### Biofilm Microscopic Analyses

A Leica DVM6 Digital Microscope was used to collect pictures of 3D-printed slides with and without bacterial biofilms. Biofilms were grown on 3D printed slides in 50 mL Falcon tubes as described in the Bacterial Adhesion section. After 3-day incubation at 37°C (50 rpm) biofilm-containing slides were washed two times with 1 × PBS solution. The entire slide was then fixed in 3% glutaraldehyde solution overnight at 4°C. After the fixation step, the slide was washed two times with distilled water and dried in a laminar flow hood for a period of 24 h. 3D projections were made using the MountainsMap^®^ ver.7.4.

### Statistical Analyses

All experiments were conducted at least in triplicates. A standard unpaired t-test was used in the case of simple two-group comparisons. Two-way analysis of variance (ANOVA) was performed on the biofilm data. MountainsMap^®^ ver.7.4. statistics module was used to analyze pictures.

## Results

### Material Characterization

#### Hydrophobicity

**FIGURE 1:**
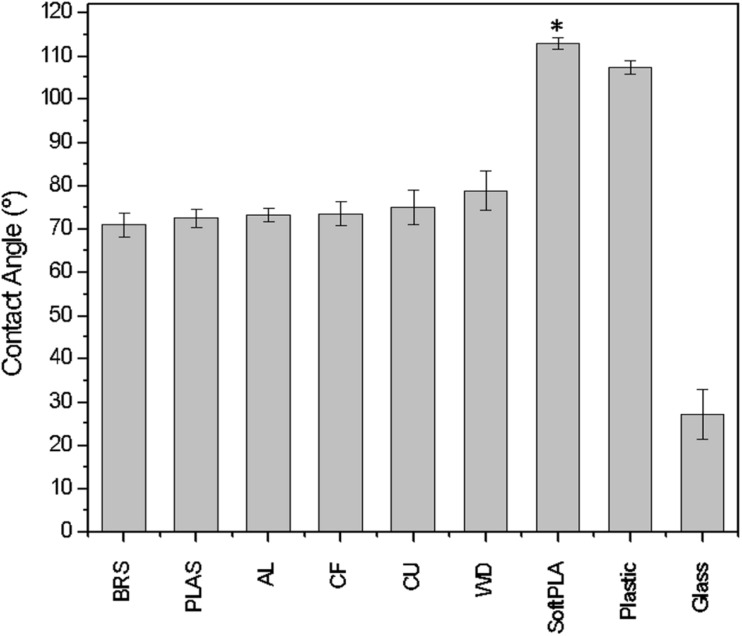
Contact angle/wettability of water droplets on 3D printouts. AL, BRS, BRZ, CF, CU, PLAS, SoftPLA, WD (Table 1). Controls are glass and plastic microscope slides. Statistical difference with other PLA polymers (^∗^).

Eight different PLA 3D materials (Table 1) were used for analyzing their bacterial biofouling properties. Some of them were supplemented with metals such as copper (CU), brass (BRS), bronze (BRZ), and aluminum (AL). These polymers were chosen as some of these metals (CU, BRS, and BRZ) have a long history of reported antimicrobial properties (Dollwet, 1985; Grass et al., 2011). The SketchUP Pro 2018 designed slides stereolithography files (STL) were printed using a MakerBot Replicator2 3D printer. To characterize the material hydrophobicity, we measured surface free energy using the OCA-15EC optical contact angle measurement system (Dataphysics). The contact angles of water droplets were measured and compared among all polymers (Figure 1). All printed materials showed higher contact angles than the glass slide control (Figure 1). Most of the polymers except SoftPLA (112.9° ± 1.34°) showed similar contact angles (74.07° ± 2.7°). SoftPLA also showed higher hydrophobicity than plastic (Figure 1).

### Surface Analyses

#### Contact Profilometry

**FIGURE 2:**
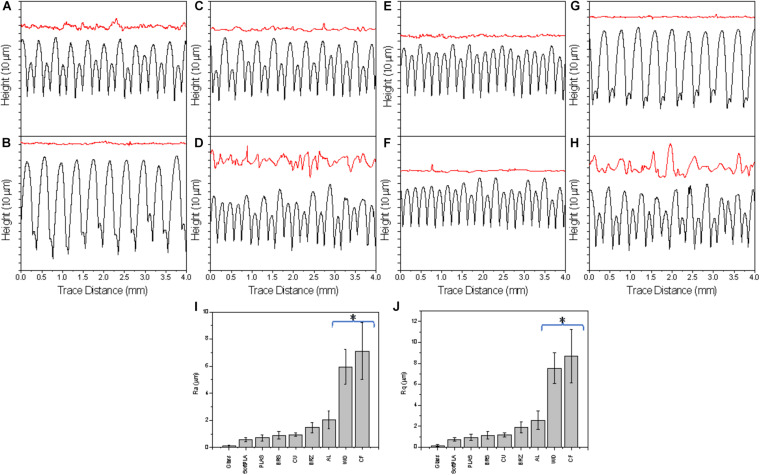
The surface profiles of 3D printed slides perpendicular (black, bottom) and parallel (red, top) the layers (representative of 4 measurements); Selected R parameters: Ra **(I)** and Rq **(J)** of surface along the 3D printouts. Polymers were ranked by ascending values. A glass microscope slide was used as a control. AL **(A)**, BRS **(B)**, BRZ **(C)**, CF **(D)**, CU **(E)**, PLAS **(F)**, SoftPLA **(G)**, WD **(H)**. (*) Statistically different group.

To characterize the surfaces of the printed slides, we first used a Mitutoyo JS210 contact profilometer. The standard unit is equipped with a 5-μm radius stylus tip, which contacts the surface with 4-mN measuring force and measures 39 different parameters. For each printout, we conducted two kinds of measurements: perpendicular (Figure 2, black) and parallel (Figure 2, red) with the extruded layers. The graphical representation clearly showed differences in both profiles. In the cross-section, each polymer showed a clear and defined profile (Figure 2, black).

Analyzing the cross-section profiles (Figure 2, black), we concluded that brass (BRS) (B) and SoftPLA (G) showed a high degree of similarity. A second pair was formed by aluminum (AL) (A) and bronze (BRZ) (C). Copper (CU) (E) was similar to PLA (PLAS) (F), while wood PLA (WD) (H) and carbon fiber PLA (CF) (D) polymers showed similarity to each other. However, analysis of the selected standard R parameters did not confirm these observations (Supplementary Figure 3). Although cross-section profiles showed interesting patterns, they represent macro-structures with R parameters ranging from several to more than a hundred micrometers (Figure 2, black and Supplementary Figure 4). These profiles did not show the real microstructure of the materials, which we thought might be more important for bacterial attachment and biofilm formation. Better, more relevant micro-profiles were obtained analyzing patterns parallel with the printing (Figure 2, red). Using R parameters of the profiles, we ranked polymers and found that the four metal-filled polymers were grouped in the middle, while “pure” PLA polymers showed lower R values. On the other hand, both WD and CF polymers showed much higher, statistically significant R values, indicating their much more complex surface structures (Figures 2I,J).

#### Non-contact Profilometry

**FIGURE 3:**
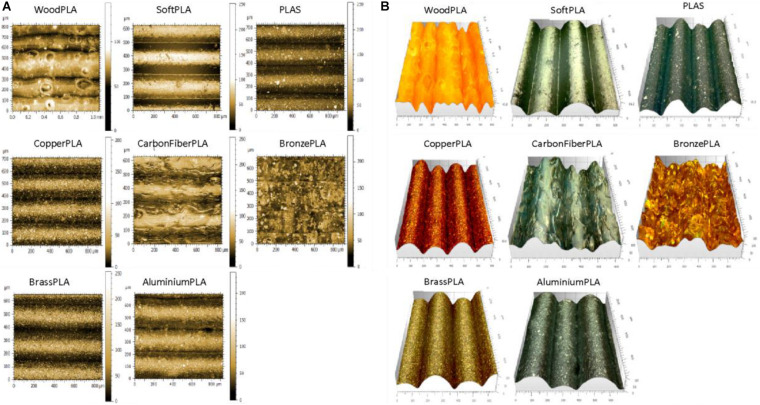
Digital microphotography of 3D printouts **(A)** and their 3D projections **(B)**.

A Leica DVM6 3D digital microscope was utilized for surface profiling of 3D printed slides. Original pictures (Figure 3A) were taken, and 3D projections were modeled and analyzed using Mountains Map software (Figure 3B). Optical profilometry revealed the true picture of the printouts. We noticed distinct structures on the surface such as knots on the WD polymer, and fibers on the CF polymer, flakes of metal on CU, BRS, BRZ, and AL, and dye in the PLAS polymer (Figures 3A,B). There were slight difficulties in imaging and 3D projecting materials that are translucent or reflect light such as wood, carbon fiber, and bronze.

Analyzing parameters calculated by the MountainsMap^®^ software, we noticed that all R parameters showed lower values compared with those obtained with the contact profilometer (Supplementary Figure 5). Also, the standard deviation values were higher in the case of the optical profilometer.

In addition to linear (2D) R parameters, optical profilometry allows for calculation of areal (3D) parameters (Gadelmawla et al., 2002; Blateyron, 2006). Surface texture parameters have a prefix that is the capital letter S (Blateyron, 2013; Pagani et al., 2017). There are many S parameters which can be divided into height parameters (Sq, Ssk, Sku, Sp, Sv, Sz, Sa), functional parameters (Smr, Smc, Sxp), amplitude parameters (Sa, Sq, Sz, Ssk, Sku, Sp, Sv, St), area and volume parameters (Smr, Sdc), 3D parameters (St, Sp, Sv, Sq, Sa, Ssk, Sku, SWt) are precisely defined by ISO, EUR, and ASME (International, European and American Organizations) (Deltombe et al., 2014). Using these parameters, we have shown that each of the 3D printouts had specific and unique characteristics (Supplementary Table 2).

### Biological Effects

#### Effect of Polymers on Bacterial Growth

**FIGURE 4:**
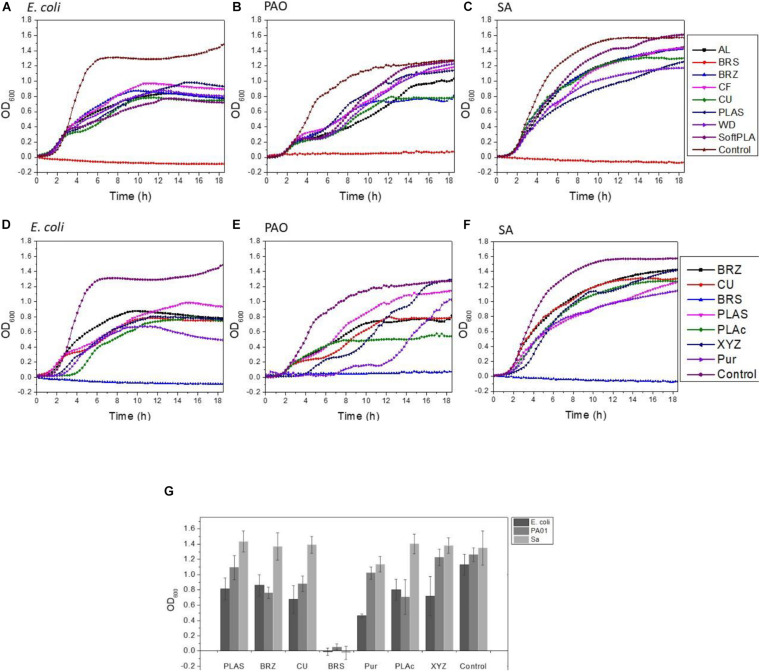
Effect of polymers on bacterial growth **(A–C)** and comparison of antimicrobial properties **(D–G)** of tested polymers. **(A,D)** *E. coli* C **(B,F)** *P. aeruginosa* PA01, **(C,F)** *S. aureus* ATCC 25923. **(G)** Number of bacteria (OD_600_) after 24-h growth with tested antimicrobial polymers and selected controls. Data obtained from 3-6 experiments with printed rings.

To analyze the effect of the 3D polymers on microorganisms, we used 3 different bacterial species: *Escherichia coli* strain C (Król et al., 2019), *Pseudomonas aeruginosa* strain PA01, and *Staphylococcus aureus* ATCC 25923. These species represent major groups of human skin and hair colonizers (Kerk et al., 2018) and in some conditions may turn into opportunistic or major pathogens (Chiller et al., 2001; Cogen et al., 2008). 3D printed rings were sterilized and loaded into 24-well plates with bacterial cultures (Supplementary Figures 1B,D). Analyzing bacterial growth, we noticed that all the polymers induced reduction in bacterial growth (Figure 4A-C). The greatest bacteriostatic effect was observed in the case of the *E. coli* strain where all polymers showed statistically significant reductions of growth (Table 2). Similar changes in the growth curves were also observed for *P. aeruginosa* and *S. aureus* (Figures 4B,C). All but the PLAS, WD, and SoftPLA polymers had statistically significant effects on growth of *P. aeruginosa*, but in the case of *S. aureus* only the BRS showed a significant effect on cell density after 24 h of growth (Table 2).

**TABLE 2:** Bacterial cell density (OD_600_) measured after 24 h of growth at 37°C.

**Polymer**	***E. coli* C**	***P. aeruginosa* PA01**	***S. aureus* ATCC 25923**
AL	0.82 ± 0.14*	0.98 ± 0.07*	1.31 ± 0.18
BRS	−0.00925 ± 0.047*	0.05 ± 0.04*	−0.02 ± 0.086*
BRZ	0.86 ± 0.14*	0.76 ± 0.075*	1.37 ± 0.18
CF	0.66 ± 0.067*	0.96 ± 0.19*	1.46 ± 0.13
CU	0.67 ± 0.18*	0.88 ± 0.1*	1.39 ± 0.11
PLAS	0.81 ± 0.14*	1.09 ± 0.16	1.43 ± 0.14
SoftPLA	0.75 ± 0.24*	0.99 ± 0.2	1.30 ± 0.15
WD	0.64 ± 0.18*	1.15 ± 0.17	1.42 ± 0.15
Control	1.13 ± 0.14	1.26 ± 0.08	1.35 ± 0.22

Data represent average values from 5-6 independent experiments. Statistically significant *p* < 0.05 (*) differences to the control.

### Biofilm Formation: Bacterial Attachment to Polymers

#### Biofilm Formation on 3D Printed Slides

Bacterial attachment is the first step in biofilm formation (Feng et al., 2015). There is increasing evidence that bacterial attachment and subsequent biofilm formation are significantly impacted by surface topography. For surfaces with topographic features at the micrometric scale, comparable with the size of prokaryotic cells, cells tend to position themselves such that they maximize contact area with the surface, which favors attachment (Mitik-Dineva et al., 2009; Bixler Gregory and Bhushan, 2012; Kostakioti et al., 2013).

**FIGURE 5:**
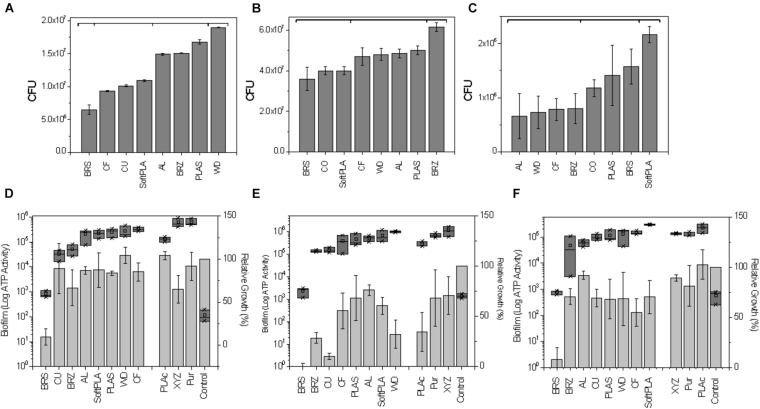
Effect of polymers on bacterial attachment after 2-h attachment **(A–C)** and biofilm formation on 3D printed pins **(D–F). (A,D)** *E. coli* C **(B,E)** *P. aeruginosa* PA01, **(C,F)** *S. aureus* ATCC 25823. **(A–C)** Number of bacterial CFU after 2 h attachment to 3D printed slides (see Supplementary Figure 2 for graphical protocol). Statistically different groups (*p* ≤ 0.05) marked by horizontal brackets. **(D–F)** Relative cell densities to pinless control (100%) are represented by gray bars. Amount of biofilm as the total ATP amount from lysed biofilm cells attached to pins are presented as dark gray blocks (Log_10_). Polymers are ranked by amount of biofilm. Data represents an average from 3 replicates.

Printed 3D slides were submerged in bacterial cultures of *E. coli* C (approximately 1.58 × 10^9^), *P. aeruginosa* PA01 (1.95 × 10^9^), and *S. aureus* ATCC 25823 (1.99 × 10^9^). After 2 h, approximately 0.01% to 3% of the initial bacteria were found attached to the surface (Figures 5A-C). In the case of *E. coli*, polymers were grouped based on the efficiency of attachment into 4 statistically significant groups (Figure 5A). The first group contained BRS with approximately 6.5 × 10^6^ attached cells. The second group with 9.3 × 10^6^ to 1.09 × 10^7^ attached cells consisted of CF, CU, and SoftPLA. The differences between polymers within that group were also statistically significant. The third group with 1.49 to 1.67 × 10^7^ bacteria contained the AL, BRZ, and PLAS polymers although both AL and BRZ are statistically different from PLAS. The final group was represented by the WD polymer with approximately 1.9 × 10^7^ attached cells which is statistically different from all other polymers.

*Pseudomonas aeruginosa* showed the highest number of attached bacteria. In this case, the polymers could be divided to 3 groups (Figure 5B). The first group contains BRS, CU, and SoftPLA (3.6 to 4.0 × 10^7^). The second group consists of the CF, WD, AL, and PLAS polymers with 4.7 to 5.0 × 10^7^ attached bacteria. In this group, only the 2 latter polymers showed statistically significant differences compared to the first group. BRZ polymer formed the last group (6.15 × 10^7^), which was statistically different from the others.

*S. aureus* showed the lowest attachment levels to the polymers in this experiment, with only 0.15% to 1% of initial bacteria attached. Three groups of polymers could be distinguished (Figure 5C). The first group with AL, WD, CF, and BRZ showed 6.6 to 7.9 × 10^5^ attached cells. In the second group with CU, PLAS and BRS polymers and almost 2 times more attached bacteria (1.2 to 1.6 × 10^6^), only the latter showed statistically significant differences as compared to the first group. The SoftPLA formed the last group with statistically significantly more attached cells (2.16 × 10^6^).

#### Biofilm Formation on 3D Printed Pins

To test biofilm using a semi-high-throughput method, the 3D pins were designed to fit a standard 48-well plate (see Supplementary Figure 1 & “Materials and Methods”). Pins were incubated with the tested bacteria for 3 days and then optical cell density of bacterial cultures were measured in the wells and amount of bacteria attached to the pins was tested by the BacTiter-Glo^TM^ Microbial Cell Viability Assay (Figures 5D-F). Pins submerged in the sterile media were used as controls.

For all 3 tested bacterial species, the least amount of biofilm formation with the lowest corresponding cell density was found in the case of the BRS polymer (Figures 5D-F). For *P*. *aeruginosa* and *S. aureus*, the next most inhibitory polymer was BRZ and it was ranked 3^rd^ in *E. coli*. Other metal-filled polymers, CU and AL, were ranked 2nd, 3rd, and 4th and 4th, 6th, and 3rd with *E. coli*, *P. aeruginosa*, and *S. aureus*, respectively. The polymers showing highly structured surfaces such as WD (ranked 7th, 8th, and 6th) and CF (ranked 5th, 4th, and 7th) and the most hydrophobic polymer SoftPLA (ranked 5th, 7th, and 8th) were on the other side, with the highest amount of biofilm (Figures 5D-F).

#### Digital 3D Microscopy for Biofilm Analysis

**FIGURE 6:**
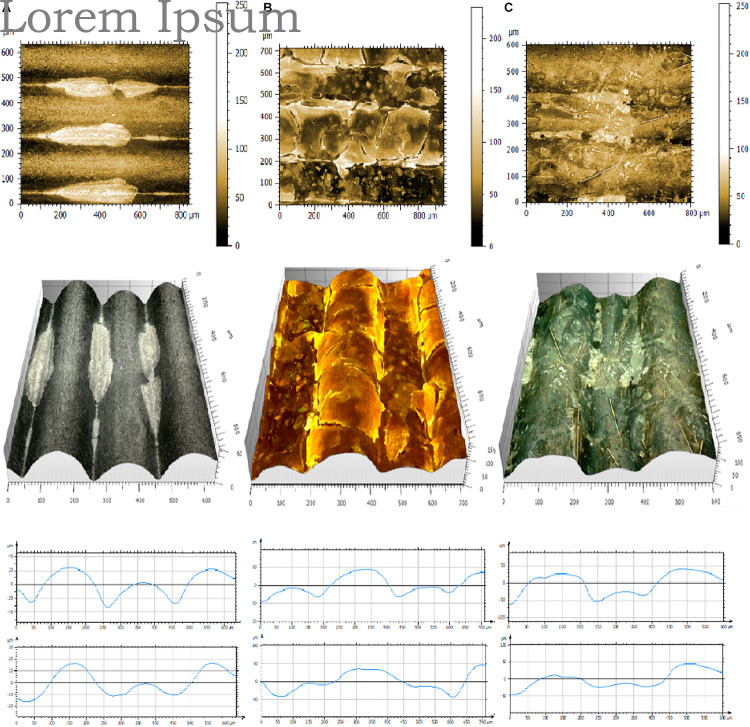
Pictures of representative biofilms formed on 3D printed materials. **(A)** *E. coli* C on SoftPLA, **(B)** *S. aureus* ATCC25823 in CU, **(C)** *P. aeruginosa* PA01 on CF. Upper panel: 2D pictures, Center panel: 3D projections, Bottom panel: projections of profiles without (upper) and with biofilms (lower) generated by MountainView software.

Microscopic observation and analysis are the most common techniques for visualizing biofilms (Lawrence and Neu, 1999). Analysis methods include standard bright field imaging though increasingly expensive epifluorescence, confocal, atomic force, and electron microscopes. To collect the data, biofilms must be prepared on special surfaces (transparent glass or plastics), treated with special dyes (CV or fluorescent dyes), or fixed, dehydrated, and covered with electron scattering heavy metals. 3D printouts are highly structured and non-transparent and therefore cannot be simply analyzed by standard microscopic techniques. We used a 3D Digital Leica DMV6 microscope to visualize biofilms attached to the 3D printed slides (Figure 6 and Supplementary Figure 6). We noticed that in most cases biofilms were at the thickest in between the layers of the 3D printed materials (Figure 6A). In the second mode of growth, the entire surface was covered by bacterial biofilm (Figure 6B). Finally, we observed that in some settings bacteria formed bridges between the highest parallel layers (Figure 6C). Using 3D projections and MountainView, we observed that generally bacterial biofilm growth tended to make the 3D printed surface smoother, i.e., the bacteria fill in the valleys (Figure 6, bottom panel).

### Comparison of Antimicrobial Properties of Studied Polymers With Commercially Available Antimicrobial Polymers

In this work, we noticed that some of the tested polymers showed bacteriostatic effect on microbes. Recently, 3 antimicrobial PLA polymers have been released to the market: Purement Antimicrobial PLA, Copper 3D PLActive Antibacterial Nanocomposite, and YXZPrinting Antibacterial PLA (Table 1). All of these materials claim to be antibacterial. PLActive has U.S. Food and Drug Administration and European Union certificates, as well as results of studies from laboratories in the United States and Chile claiming the bactericidal effect against *E. coli* DH5α and an *S. aureus* MRSA strain^1^. XYZPrinting PLA is impregnated with silver ions as the antimicrobial compound. These silver ions create reactive oxygen species according to the manufacturer. PLActive Copper 3D filament’s method of action has been described as “copper oxide nanocomposite infused PLA.” We compared the antimicrobial properties of these commercially available antimicrobial polymers to those of our test polymers.

In growth curve experiments, we noticed no difference between the PLActive polymer and normal PLAS (Figures 4D-F). Growth kinetics for XYZPrinting and Purement Antimicrobial polymers depended greatly on the bacterial strain. In the case of *E. coli* C, we saw a slight delay in growth and low final cell density (Figures 4D,G). However, the differences in cell densities between Purement and PLAS or BRZ were statistically significant, but they were not significantly different from the third tested polymer, CU (Figure 4G). In *P. aeruginosa*, both polymers showed a 2- to 4-h delay in growth; however, the final cell density after 17 h was not affected (Figures 4E,G). In the case of S. *aureus*, only a slight lag phase was observed, with no significant changes in final cell densities (Figures 4F,G). None of the tested antimicrobial polymers could compare with BRS in terms of its antimicrobial properties (Figure 4).

Next, we compared biofilm formation on the antimicrobial polymers and our previously tested ones (Figures 5D-F). In the case of *E. coli* C, the PLActive showed the least amount of biofilm formation, which was statistically significant compared to BRS, CU, and BRZ. Both the XYZPrinting and Purement polymers showed the highest amounts of biofilm formation and were statistically higher than PLActive, AL, SoftPLA, and PLAS but not the WD and CF polymers (Figure 5D).

We showed that the BRS, BRZ, and CU polymers were the most inhibitory for *P. aeruginosa* biofilm formation. Tests with the three commercial antimicrobial polymers showed that none of them were more efficient in PA biofilm inhibition. Again, the least amount of biofilm formed was observed on the PLActive polymer; however, it was not statistically better than the CU polymer and only slightly better than the BRZ polymer (Figure 5E). The amount of biofilm on the PLActive polymer was statistically less only when compared to WD. Purement also did not prevent biofilm formation and resulted in more biofilm growth than all test polymers except WD (*p* = 0.03); BRS, BRZ. and CU were all statistically better than the Purement polymer for inhibiting PA biofilm growth. The XYZPrinting antimicrobial polymer showed the highest amount of *P. aeruginosa* biofilm; however, statistical differences again were observed only with BRS, BRZ, and CU (Figure 5E).

The smallest differences in biofilm formation on 3D polymers were observed in the case of *S. aureus* (Figure 5F) which also was the poorest of the bacterial species in the adhesion experiments. The least amount of biofilm was formed on BRS and the highest on SoftPLA. Both polymers were statistically different from the others except SoftPLA and PLActive.

To conclude, none of the commercial antimicrobial polymers were better than our metal-filled polymers BRS, BRZ, and CU and the differences between the remaining polymers were not really striking.

## Discussion

Since its development in the early 1980s, 3D printing technology has become ever more popular in many fields of science and industry (Liaw and Guvendiren, 2017; Ngo et al., 2018; Hao and Lin, 2020). At the same time, the development of antibiotic-resistant bacterial pathogens is a real threat in the healthcare industry (Lin et al., 2015). Biofilms, surface-attached bacterial aggregation, have been found to be 1000 × more resistant to antibiotics than their planktonic counterparts (Mah and O’Toole, 2001; Hall-Stoodley et al., 2008). Biofilms have been noted as emerging problems in medicine, especially on medical devices and implants (Braxton et al., 2005; Ehrlich et al., 2005a,b, 2014; Stoodley et al., 2005; Fux et al., 2006; Wolcott and Ehrlich, 2008; Nowatzki et al., 2012; Palmer et al., 2014; Srivastava et al., 2019). During the current COVID-19 pandemic, the shortage of personal protection equipment (PPE) enforced the usage of 3D printed technology to produce face shields and facemasks (Flanagan and Ballard, 2020; Swennen et al., 2020). Because of the increased use of 3D printing in the medical fields, we decided to take a closer look at the properties of available polymers and their interactions with common biofilm-forming bacteria. PLA polymers were our main focus as they are made from natural resources, can be enzymatically degraded, and are safe for humans (Conn et al., 1995).

Several of our tested polymers have been filled with metals/alloys such as copper, brass, bronze, and aluminum. Copper, brass, and bronze have a long history of being used as antimicrobial agents (Dollwet, 1985; Grass et al., 2011) while aluminum has no or limited effect on bacterial growth (Varkey, 2010). PLA with carbon fibers and wood represented a second group of polymers with more structured surfaces, while PLA with silver dye was used as a PLA-only control. We used optical contact angle measurements to analyze the hydrophobicity of the prints and both surface contact and optical profilometry to analyze their surface. While hydrophobicity measurements were very straight forward, we faced some problems using both surface and optical profilometers. It was noted that all cross-section profiles tested by the Mitutoyo JS210 contact profilometer gave distinct patterns, but their lines were relatively smooth (Figure 2, black). That indicated that the big structural changes across the 3D printout hid the true micro-surface properties, which were revealed by along-layer profile measurements (Figure 2, red; Figures 2I-L). We also noticed that although the printer layer height was set to 200 μm, the R_Sm_ (mean peak width) parameter showed a large variation from 156 μm to almost 400 μm, indicating a limited precision of contact profilometry (Supplementary Figure 4).

Optical profilometry is a rapid, non-destructive, and non-contact surface metrology technique. An optical profiler is a type of microscope in which light from a lamp is split into two paths by a beam splitter and each light beam is used in forming topographic information (Wang et al., 2017). The optical profilometers have been used in material science and industry for decades. A different kind of 3D microscopy was used to analyze biological materials. These methods used expensive and sophisticated equipment like confocal microscopy (Lawrence and Neu, 1999; Palmer and Sternberg, 1999). Unfortunately, old profilometers (like Zygo^®^) and confocal microscopes are too precise or require fluorescent dyes and cannot really be used in the case of 3D printouts. Recent progress in imaging technology allowed construction of 3D digital optical microscopes such as Zeiss SmartZoom 5 (Zeiss, United States), Hirox RH-8800 (Hirox, United States), Keyence VR-5000, and VHX-7000 (Keyence, United States) and Leica DVM6 (Leica, United States). These microscopes combine a high magnification (up to 7,000×) with high resolution and sophisticated software to provide 3D capabilities. We have tested some of these instruments and found Leica, Keyence, and Hirox most suitable for our studies (Supplementary Figures 6–8). Unfortunately, 3D digital microscopy faces some problems. 3D projecting materials that are very dark, translucent, or reflect light, such as wood, carbon fiber, and bronze, are hard to image using 3D digital microscopy (see Figure 3 and Supplementary Figures 6–8). Even if the pictures represent the true structures, 3D projections and surface R and S parameters do not always reflect it. More work must be done to improve this technique, such as coatings to reduce glare and lighting issues.

Three common human-associated bacterial species (*E. coli* strain C, *P. aeruginosa* PA01, and *S. aureus* ATCC25823) were employed to test the effect of 3D printouts on growth, attachment, and biofilm formation. Using 24-well plates with 3D printed rings we were able to test relatively fast and simply the effect of polymers on planktonic growth, showing that almost all polymers affect the growth. The differences might be attributed to the bacteriostatic properties of the polymers, as well as to the increased surface area, which sequesters some planktonic cells. We believe these sequestered cells are part of the first step of biofilm formation (Costerton et al., 1995; Donlan, 2002; Bixler Gregory and Bhushan, 2012; Feng et al., 2015). Two hours of interactions between printouts and 10^9^ bacterial cells was not enough to kill the bacteria or reduce the number of bacteria in the culture as we observed in the growth experiments. Therefore, attachment should be affected by other parameters, i.e., surface roughness and hydrophobicity (Merritt and An, 2000; Mitik-Dineva et al., 2009). To analyze bacterial attachment, we used 3D printed slides partially submerged in the water. As adhesion to the surface is higher at the air-liquid interface we used this model to get a better idea of these complex interactions between bacteria and 3D printouts.

In the case of *E. coli*, the highest number of attached cells was observed on the wood polymer, which was ranked the second-most rough material (Figures 2, 5). In the case of *P. aeruginosa*, the correlation between material roughness and cell attachment is not that straightforward. We speculate that *Pseudomonas*, with its “slime” and other extracellular appendages, overcame those surface parameters and maybe used its own factors for successful attachment (Chang, 2018). In the case of *S. aureus*, the highest number of attached cells was observed in the case of SoftPLA, which showed the smoothest surface of all tested polymers (Figure 2). On the other hand, the hydrophobicity of the SoftPLA surface was by far the highest of the tested polymers (Figure 1). Recently, Forson and colleagues showed that *S. aureus* has a much higher attachment rate to silanized glass (water contact angle, 96° ± 8°) than substrates with lower hydrophobicity (Forson et al., 2020).

While analyzing biofilm formation on 3D printouts, we realized that 3 principal parameters (antibacterial properties, surface structure, and hydrophobicity of the polymers) play important roles. All 3 (or 4) metal-filled polymers with their antimicrobial properties showed the least amount of biofilm formed for all 3 bacteria species (Figures 5D-F). On the other hand, most rough surfaces (carbon fiber and wood) were almost always the most supportive of biofilm growth. Additionally, in the case of *P. aeruginosa* and more importantly *S. aureus*, high surface hydrophobicity (SoftPLA) played the major role in biofilm formation.

In the biofilm image analyses, it was not surprising that we observed that most of the biofilms filled the grooves between printed layers. In a several cases, the biofilms formed bridges between higher layers. *P. aeruginosa* and *S. aureus* covered the surfaces more completely than *E. coli*. This observation might be related to shear force, which even at a low level (50 rpm) triggers a mechanism of finding a safe niche. The latter is related to the bacterial properties as both *P. aeruginosa* and *S. aureus* are the ultimate biofilm formers. Although, the grooves in 3D printouts can be smoothed, the process for PLA polymers is not that easy as for the ABS and other polymers and requires additional time and effort.

The antimicrobial properties of the polymers play a crucial role in reducing biofilm formation. The first antimicrobial PLA polymer with different concentrations of silver nanoparticles was developed in 2013; however, the fibers (not printouts) were tested only against planktonic cells (Erem et al., 2013). Currently, a few antibacterial polymers are available on the market. Here we tested 3 out of 5 of these polymers. The price of these antimicrobial polymers is usually 3 to 4 times higher than the standard and metal-filled polymers tested in this work. The exact composition of antimicrobial polymers is usually not revealed, and all provided information is vague. Although we noticed slight antimicrobial properties of these polymers, they were not statistically different from metal filled brass, copper, and bronze PLA polymers. The greatest antimicrobial and antibiofilm activity were observed in the case of BRS PLA polymer (Figures 4, 5D-F). According to the manufacturer, this polymer contains about 40% metal.

To the best of our knowledge, this is the first report describing biofilm formation on 3D printed surfaces, with as many as 8 standard and 3 antimicrobial PLA polymers, as well as 3 of the most prevalent human colonizers: *E. coli*, *P. aeruginosa*, and *S. aureus*. We hope that the methods described here might set some standards for developing and testing new antimicrobial polymers with different bacterial species and this research may lead to reducing the chances of bacterial infections spread by 3D printed devices.

## Data Availability Statement

The original contributions presented in the study are included in the article/Supplementary Material, further inquiries can be directed to the corresponding author/s.

## Author Contributions

DH and JK designed the study, performed the experiments and analyses, wrote the original draft, and edited the manuscript. PP performed experiments, analyzed data, and edited the manuscript. GE supported the project and edited the entire manuscript. H-FJ supported the project. All authors read and approved the final version of the manuscript.

## Conflict of Interest

The authors declare that the research was conducted in the absence of any commercial or financial relationships that could be construed as a potential conflict of interest.

## Footnotes

1 https://copper3d.com/

## Supplementary Material

Supplementary Figure 1 Designed 3D slides, rings, and pins **(A)**, rings in 24 well plate **(B)**.

Supplementary Figure 2 Schematic representation of adsorption experiment. 1: 3D slide placed in overnight culture of bacteria; 2: 2 h incubation at 37°C, 50 rpm; 3&4: washing in sterile PBS; 5: Shaking 3 min 1000 rpm on Mini-G, SPEX-Sample-Prep; 6: Dilutions were plated by drip titration method.

Supplementary Figure 3 Selected Mitutoyo R parameters on cross-sections of printouts ranked ascendant (average data from 4 measurements). Ra - Arithmetic mean deviation of the roughness profile; Rq - Root-mean-square (RMS) deviation of the roughness profile; Rz - Maximum Height of roughness profile; Rp - Maximum peak height of the roughness profile; Rv - Maximum valley depth of the roughness profile.

Supplementary Figure 4 RSm (mean peak width) parameters on cross-sections of printouts (average data from 4 measurements).

Supplementary Figure 5 Selected R parameters on cross-sections of printouts (average data from 9 measurements). Ra - Arithmetic mean deviation of the roughness profile; Rq - Root-mean-square (RMS) deviation of the roughness profile; Rz - Maximum Height of roughness profile; Rp - Maximum peak height of the roughness profile; Rv - Maximum valley depth of the roughness profile; Rc - Mean height of the roughness profile elements.

Supplementary Figure 6 Pictures of representative biofilms formed by *E. coli* C, *P. aeruginosa* PA01, and *S. aureus* ATCC25823 on 3D printed materials. Upper panel: 2D pictures, Center panel: 3D projections, Bottom panel: projections of profiles without (upper) and with biofilms (lower) generated by MountainView^®^ software.

Supplementary Figure 7 3D projections of selected printouts obtained with Keyence VHX-7000 Laser Scanning Confocal Microscope. From left top: CU, SoftPLA, WD, CF, BRS, BRZ.

Supplementary Figure 8 3D projections of selected printouts obtained with Hirox KH8700 microscope. From left top: CU, WD, SoftPLA, PLAS, BRS. Bottom: *P. aeruginosa* and *S. aureus* biofilms on CU (left and right, respectively).

Supplementary Table 1 Most commonly used surface parameters.

Supplementary Table 2 Characteristics of the 3D printouts generated by MountainMap^®^ software.
