# Supplementary material for: Bacterial Biofilm Growth on 3D-Printed Materials
Source: Front Microbiol. 2021 May 28;12:646303. doi: 10.3389/fmicb.2021.646303 (PMC8192718; doi:10.3389/fmicb.2021.646303)
Supplement: Supplementary Figure 1 — Designed 3D slides, rings, and pins (A), rings in 24 well plate (B). [file Data_Sheet_1.docx]

**Tabla S1 Most commonly used surface parameters.**

| **Abbreviation** | **Parameter** |
| --- | --- |
| Rz | Maximum height |
| Rp | Maximum profile peak height |
| Rv | Maximum profile valley depth |
| Rc | Mean height |
| Rt | Total height |
| Ra | Arithmetic mean deviation |
| Rq | Root mean square deviation |
| Rsk | Skewness |
| Rku | Kurtosis |
| RSm | Mean width |
| Rdq | Root mean square slope |
| Rmr | Material ratio |
| Rdc | Profile section height difference |
| Rmr | Relative material ratio |
| Rk | Core roughness depth |
| Rpk | Reduced peak height |
| Rvk | Reduced valley height |

**Table S2. Characteristics of the 3D printouts generated by MountainMap® software.**

| ISO 25178 | **AL** | **BRS** | **BRZ** | **CF** | **CU** | **PLA-S** | **Soft** | **WD** |  |  |
| --- | --- | --- | --- | --- | --- | --- | --- | --- | --- | --- |
| Height Parameters | |  |  |  |  |  |  |  |  |  |
| Sq | 33.2 | 25.4 | 26.2 | 24.6 | 17.2 | 20.8 | 23.6 | 12.9 | µm | Root-mean-square height |
| Ssk | -0.0737 | 0.189 | -0.173 | -0.0123 | -0.382 | 0.269 | 0.268 | -1.02 |  | Skewness |
| Sku | 1.61 | 2.11 | 5.86 | 2.33 | 2.3 | 1.88 | 1.89 | 2.66 |  | Kurtosis |
| Sp | 52.3 | 50.9 | 72.2 | 59.6 | 41 | 39.8 | 44.2 | 11.3 | µm | Maximum peak height |
| Sv | 60.5 | 61.3 | 198 | 82.9 | 42.6 | 46.9 | 42 | 32.5 | µm | Maximum pit height |
| Sz | 113 | 112 | 271 | 143 | 83.6 | 86.8 | 86.2 | 43.8 | µm | Maximum height |
| Sa | 29.7 | 21.2 | 20.3 | 20.5 | 14.2 | 17.8 | 20 | 10.9 | µm | Arithmetic mean height |
| Functional Parameters | | |  |  |  |  |  |  |  |  |
| Smr | 0.487 | 0.155 | 0.0125 | 0.08 | 0.0421 | 0.0954 | 0.362 | 26.6 | % | Areal material ratio |
| Smc | 43.5 | 38.4 | 39 | 33.3 | 20.5 | 31.4 | 35.7 | 11.2 | µm | Inverse areal material ratio |
| Sxp | 56.2 | 37.6 | 36.8 | 43.8 | 36.3 | 28.2 | 33.8 | 35.1 | µm | Extreme peak height |
| EUR 15178N | |  |  |  |  |  |  |  |  |  |
| Amplitude Parameters | | |  |  |  |  |  |  |  |  |
| Sa | 29.7 | 21.2 | 20.3 | 20.5 | 14.2 | 17.8 | 20 | 10.9 | µm | Arithmetic mean deviation |
| Sq | 33.2 | 25.4 | 26.2 | 24.6 | 17.2 | 20.8 | 23.6 | 12.9 | µm | Root-mean-square mean deviation |
| Sz | 86.3 | 51 | 127 | 98.7 | 56.1 | 53.7 | 53.4 | 26.5 | µm | Ten point height |
| Ssk | -0.0737 | 0.189 | -0.173 | -0.0123 | -0.382 | 0.269 | 0.268 | -1.02 |  | Skewness |
| Sku | 1.61 | 2.11 | 5.86 | 2.33 | 2.3 | 1.88 | 1.89 | 2.66 |  | Kurtosis |
| Sp | 52.3 | 50.9 | 72.2 | 59.6 | 41 | 39.8 | 44.2 | 11.3 | µm | Maximum peak height |
| Sv | 60.5 | 61.3 | 198 | 82.9 | 42.6 | 46.9 | 42 | 32.5 | µm | Maximum valley depth |
| St | 113 | 112 | 271 | 143 | 83.6 | 86.8 | 86.2 | 43.8 | µm | Total height |
| Area and Volume Parameters | | |  |  |  |  |  |  |  |  |
| Smr | 0.487 | 0.155 | 0.0125 | 0.08 | 0.0421 | 0.0954 | 0.362 | 26.6 | % | Areal material ratio |
| Sdc | 77.6 | 60 | 59.5 | 56.4 | 37.7 | 50.9 | 57.2 | 24.5 | µm | Areal height difference |
| ASME B46.1 | |  |  |  |  |  |  |  |  |  |
| 3D Parameters | |  |  |  |  |  |  |  |  |  |
| St | 113 | 112 | 271 | 143 | 83.6 | 86.8 | 86.2 | 43.8 | µm | Maximum height |
| Sp | 52.3 | 50.9 | 72.2 | 59.6 | 41 | 39.8 | 44.2 | 11.3 | µm | Maximum peak height |
| Sv | 60.5 | 61.3 | 198 | 82.9 | 42.6 | 46.9 | 42 | 32.5 | µm | Maximum pit height |
| Sq | 33.2 | 25.4 | 26.2 | 24.6 | 17.2 | 20.8 | 23.6 | 12.9 | µm | Root-mean-square height |
| Sa | 29.7 | 21.2 | 20.3 | 20.5 | 14.2 | 17.8 | 20 | 10.9 | µm | Arithmetic mean height |
| Ssk | -0.0737 | 0.189 | -0.173 | -0.0123 | -0.382 | 0.269 | 0.268 | -1.02 |  | Skewness |
| Sku | 1.61 | 2.11 | 5.86 | 2.33 | 2.3 | 1.88 | 1.89 | 2.66 |  | Kurtosis |
| SWt | 70.2 | 51.6 | 68.9 | 58.4 | 36.1 | 40.8 | 54.8 | 33.6 | µm | Area waviness height |


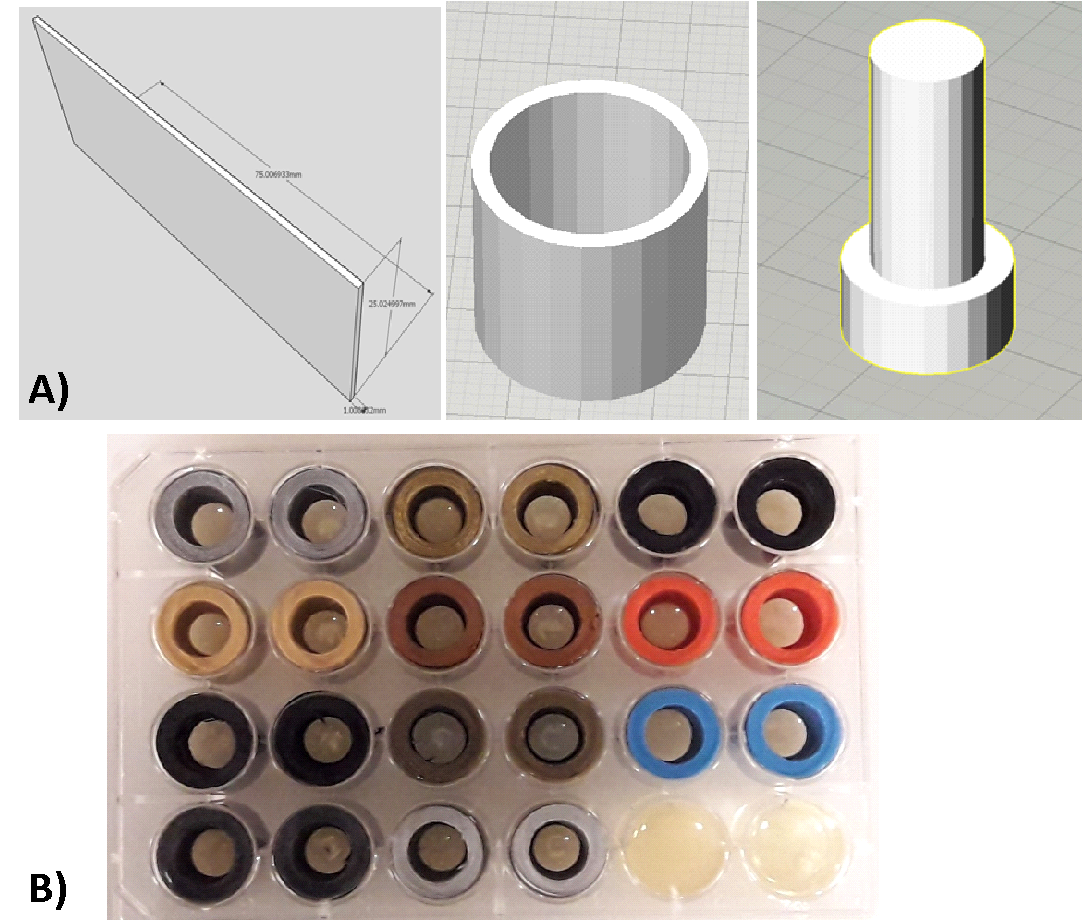


**Figure S1. Designed 3D slides, rings, and pins (A), rings in 24 well plate (B).**


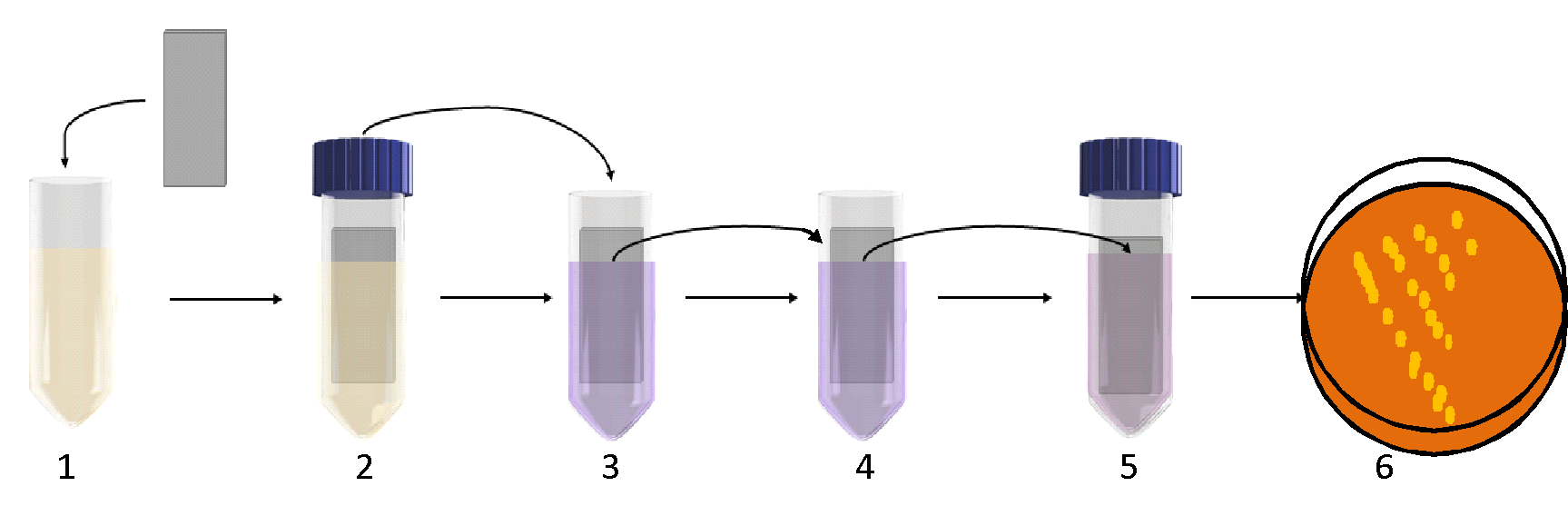


**Figure S2. Schematic representation of adsorption experiment.** 1: 3D slide placed in overnight culture of bacteria; 2: 2 h incubation at 37°C, 50 rpm; 3&4: washing in sterile PBS; 5: Shaking 3 min 1000 rpm on Mini-G, SPEX-Sample-Prep; 6: Dilutions were plated by drip titration method.


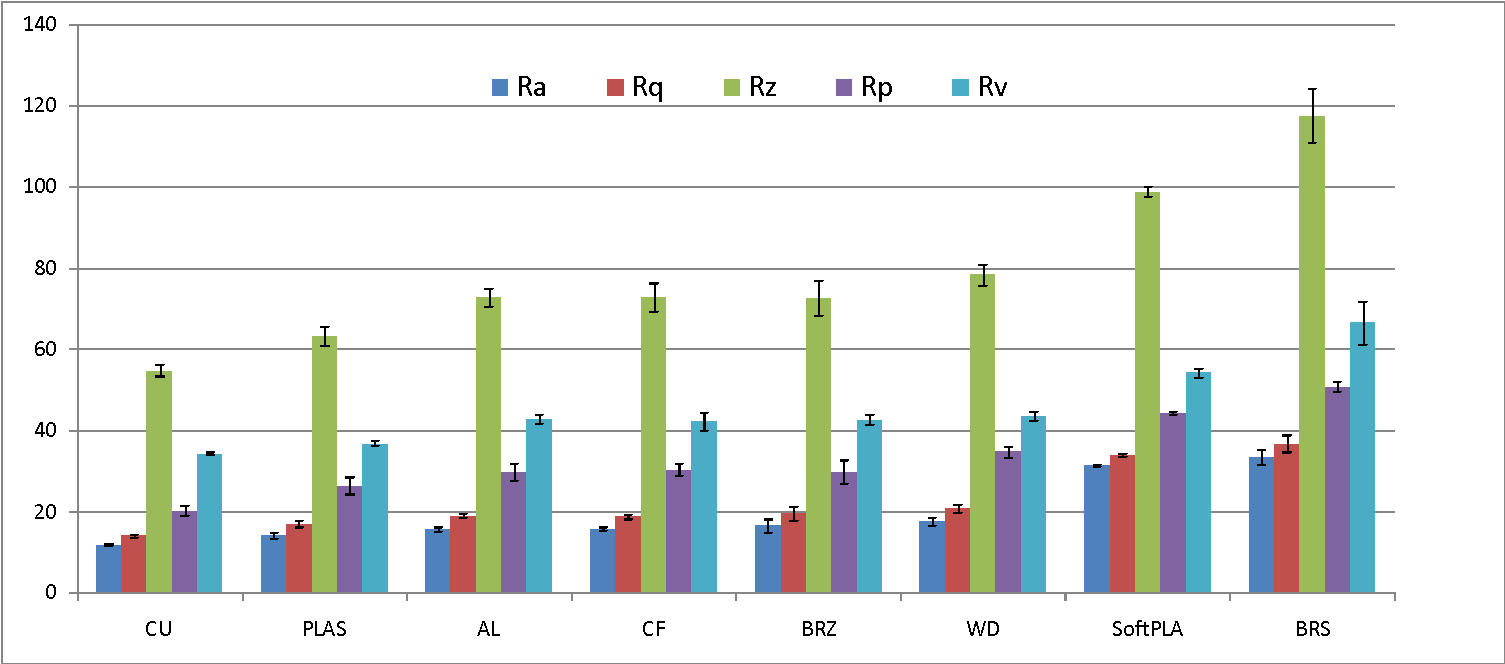


µm

**Figure S3. Selected Mitutoyo R parameters on cross-sections of printouts ranked ascendant** (average data from 4 measurements). Ra - Arithmetic mean deviation of the roughness profile; Rq - Root-mean-square (RMS) deviation of the roughness profile; Rz - Maximum Height of roughness profile; Rp - Maximum peak height of the roughness profile; Rv - Maximum valley depth of the roughness profile


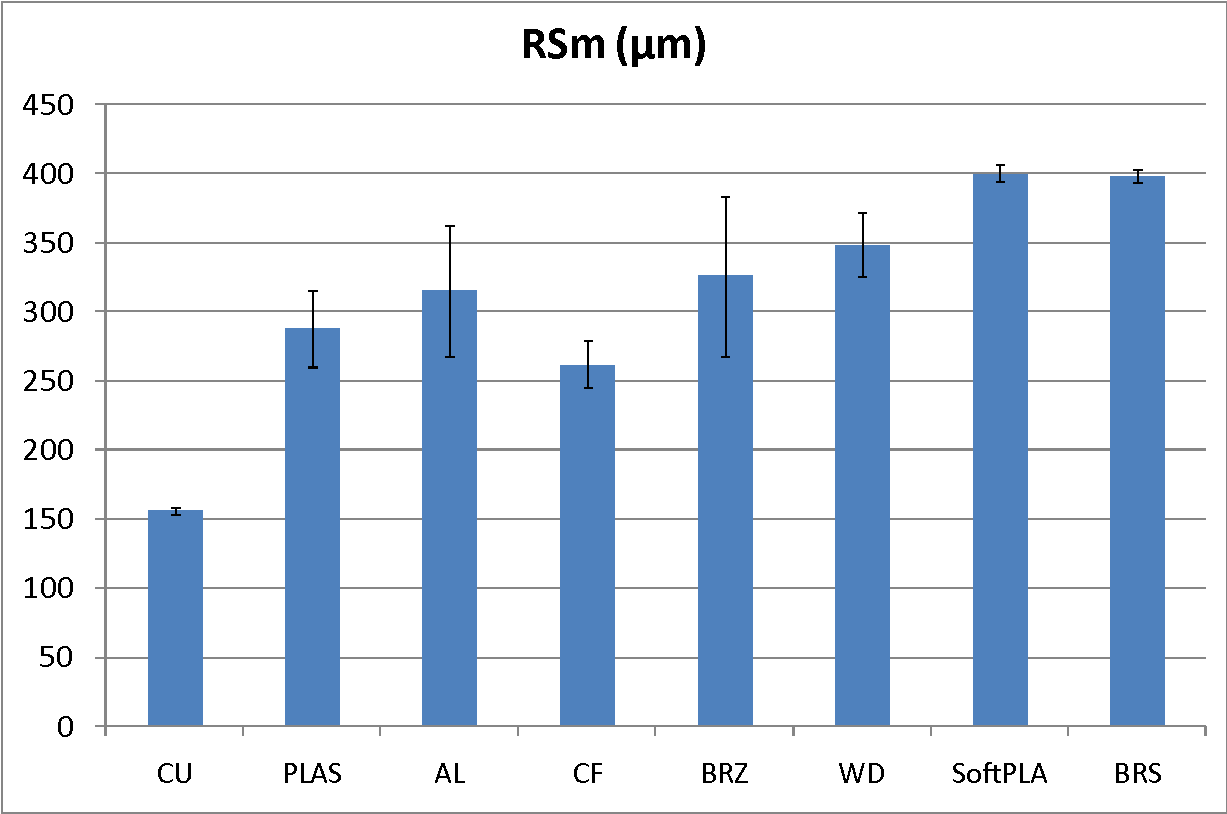


**Figure S4.** **RSm (mean peak width) parameters on cross-sections of printouts** (average data from 4 measurements).


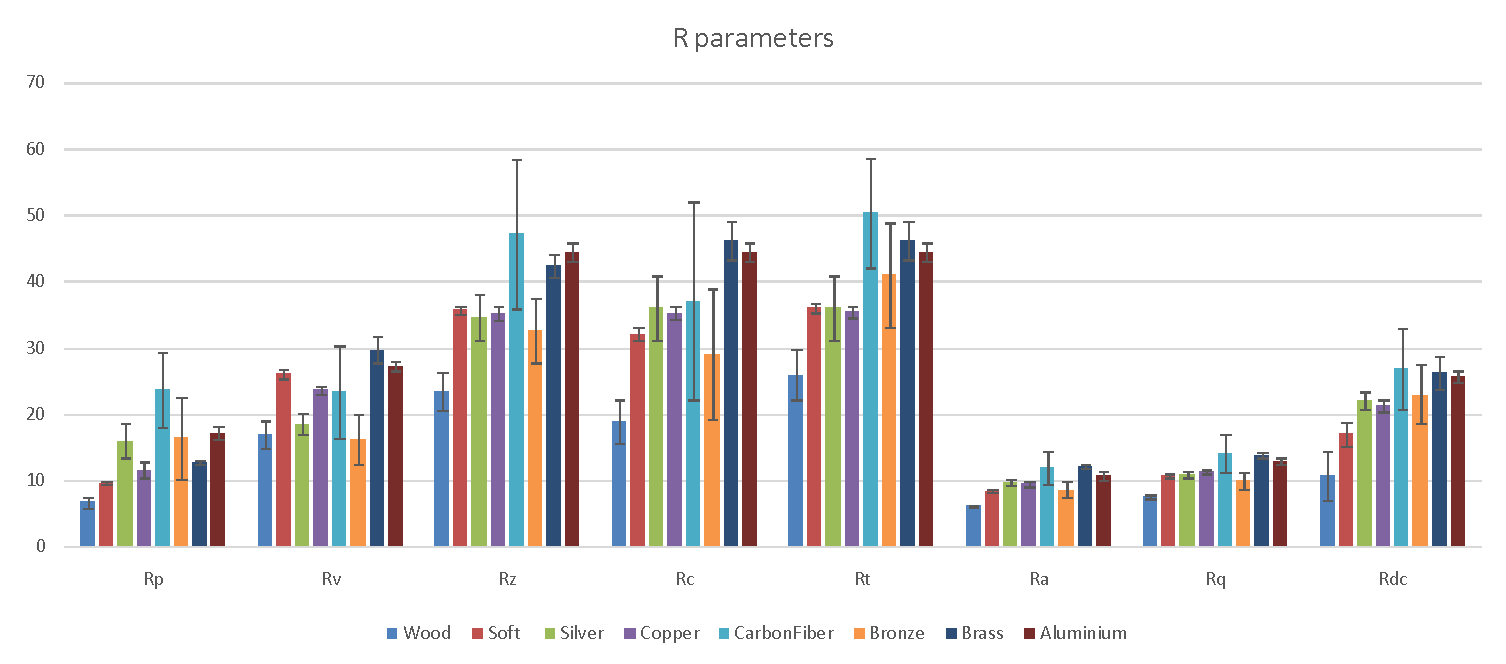


**Figure S5.** **Selected R parameters on cross-sections of printouts** (average data from 9 measurements). Ra - Arithmetic mean deviation of the roughness profile; Rq - Root-mean-square (RMS) deviation of the roughness profile; Rz - Maximum Height of roughness profile; Rp - Maximum peak height of the roughness profile; Rv - Maximum valley depth of the roughness profile; Rc - Mean height of the roughness profile elements.


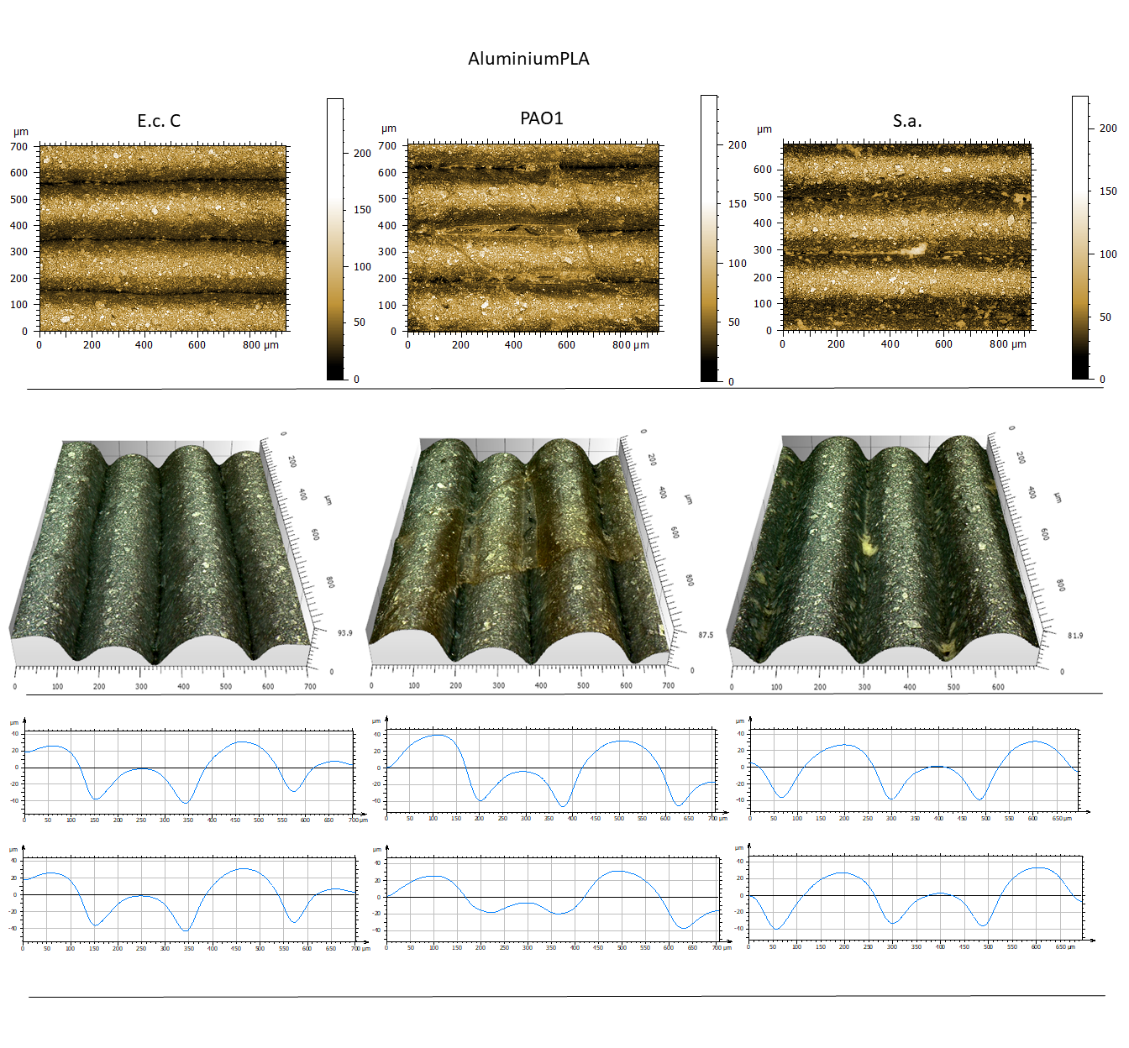


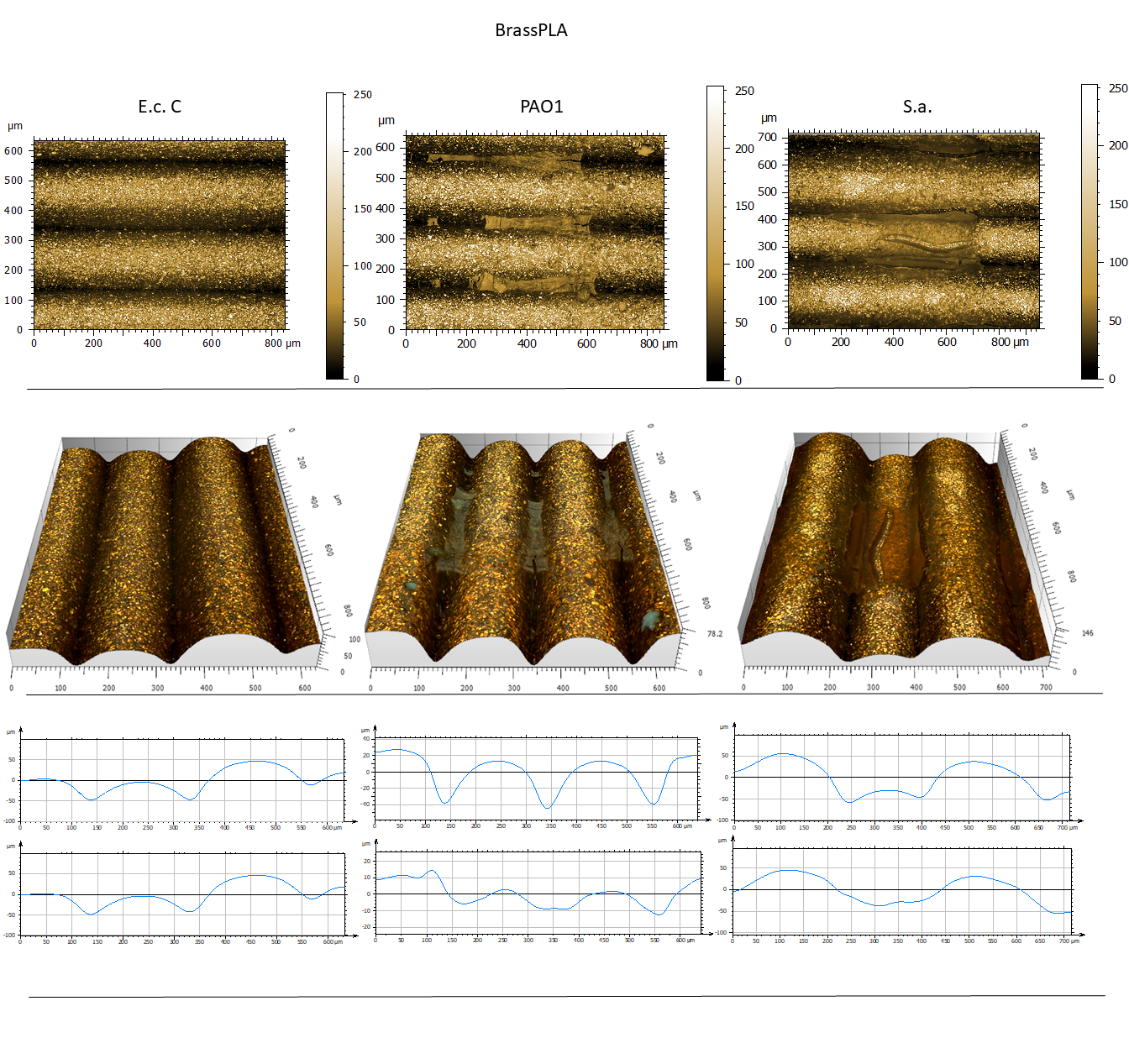


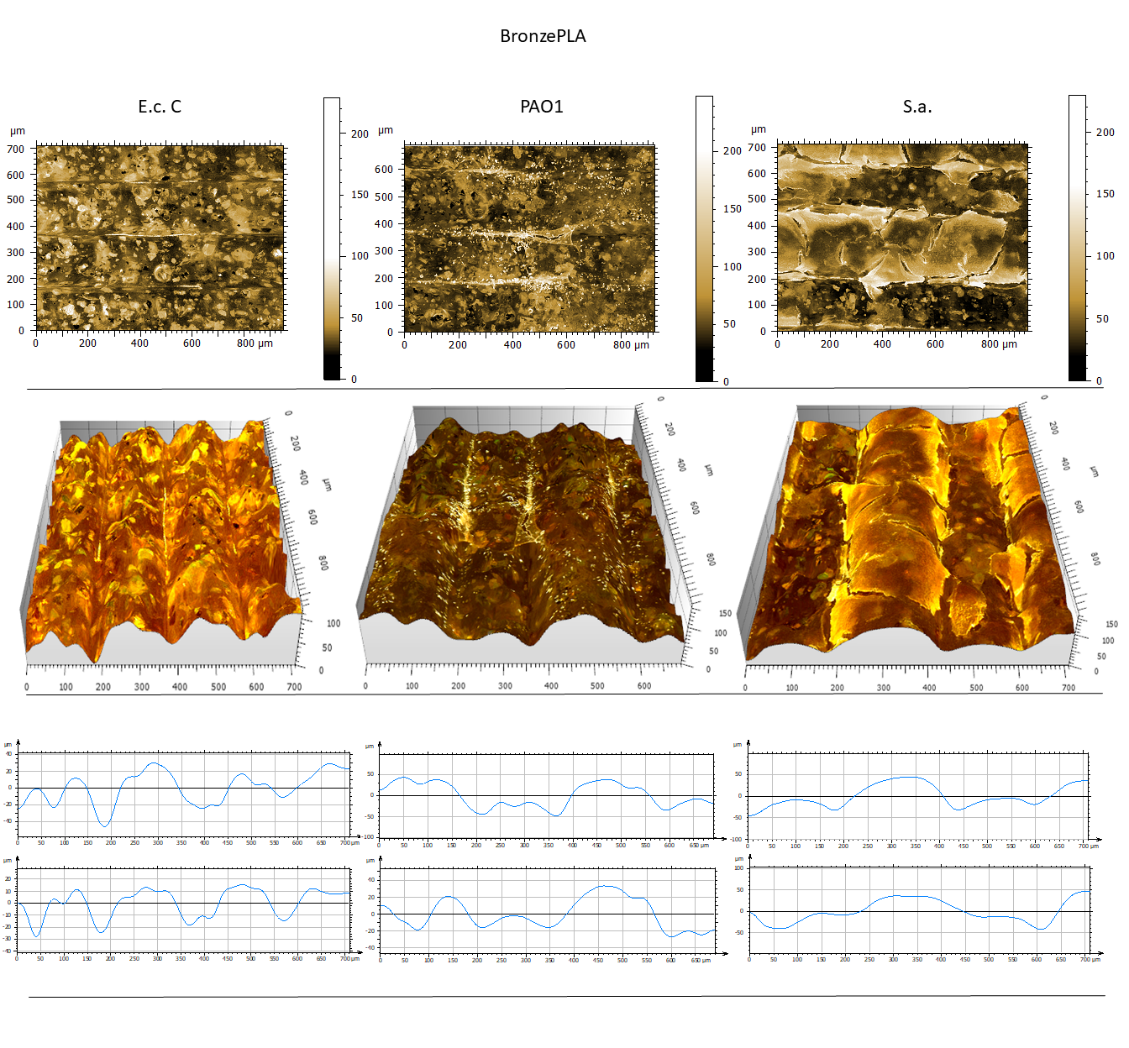


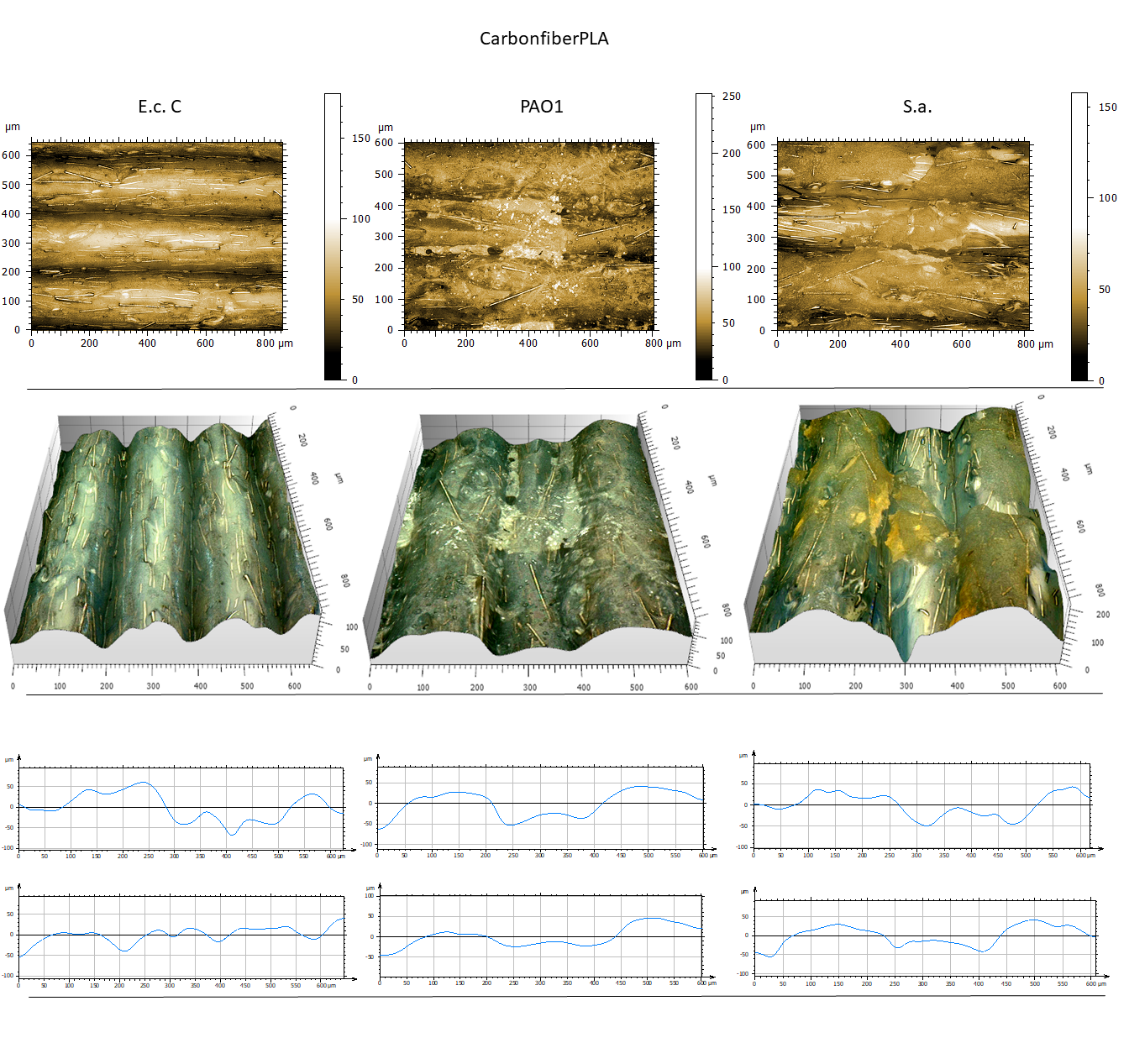


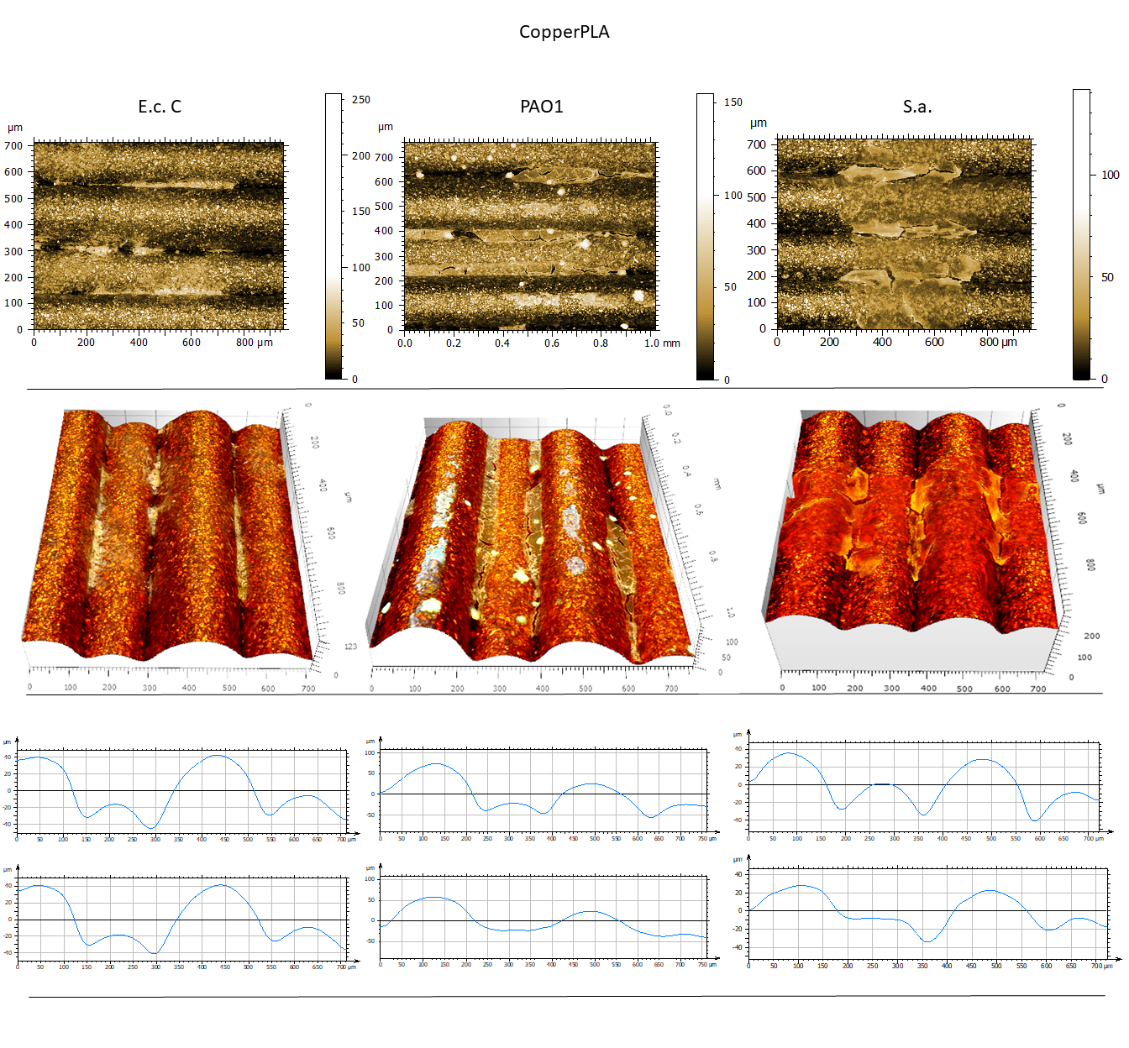


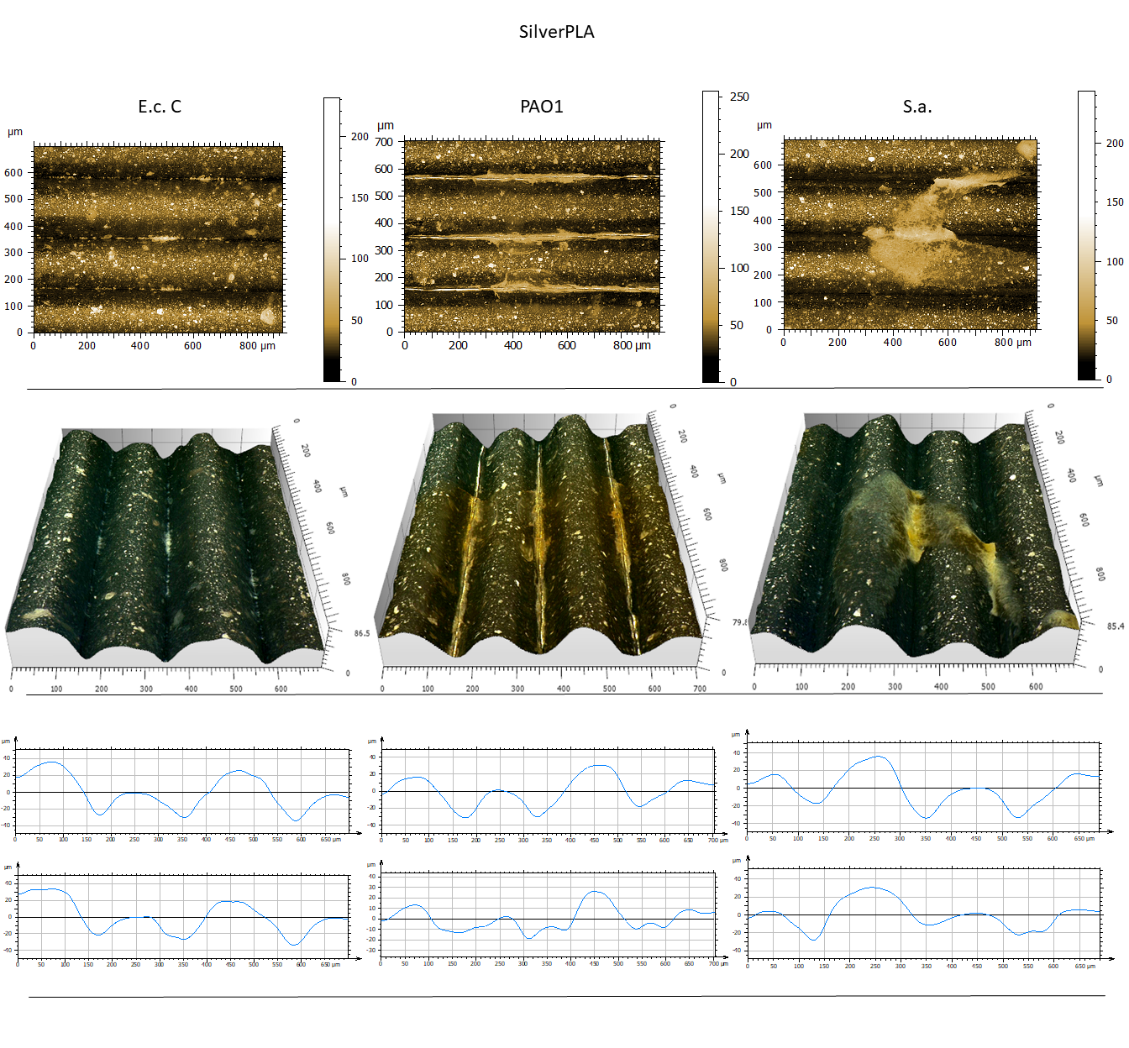


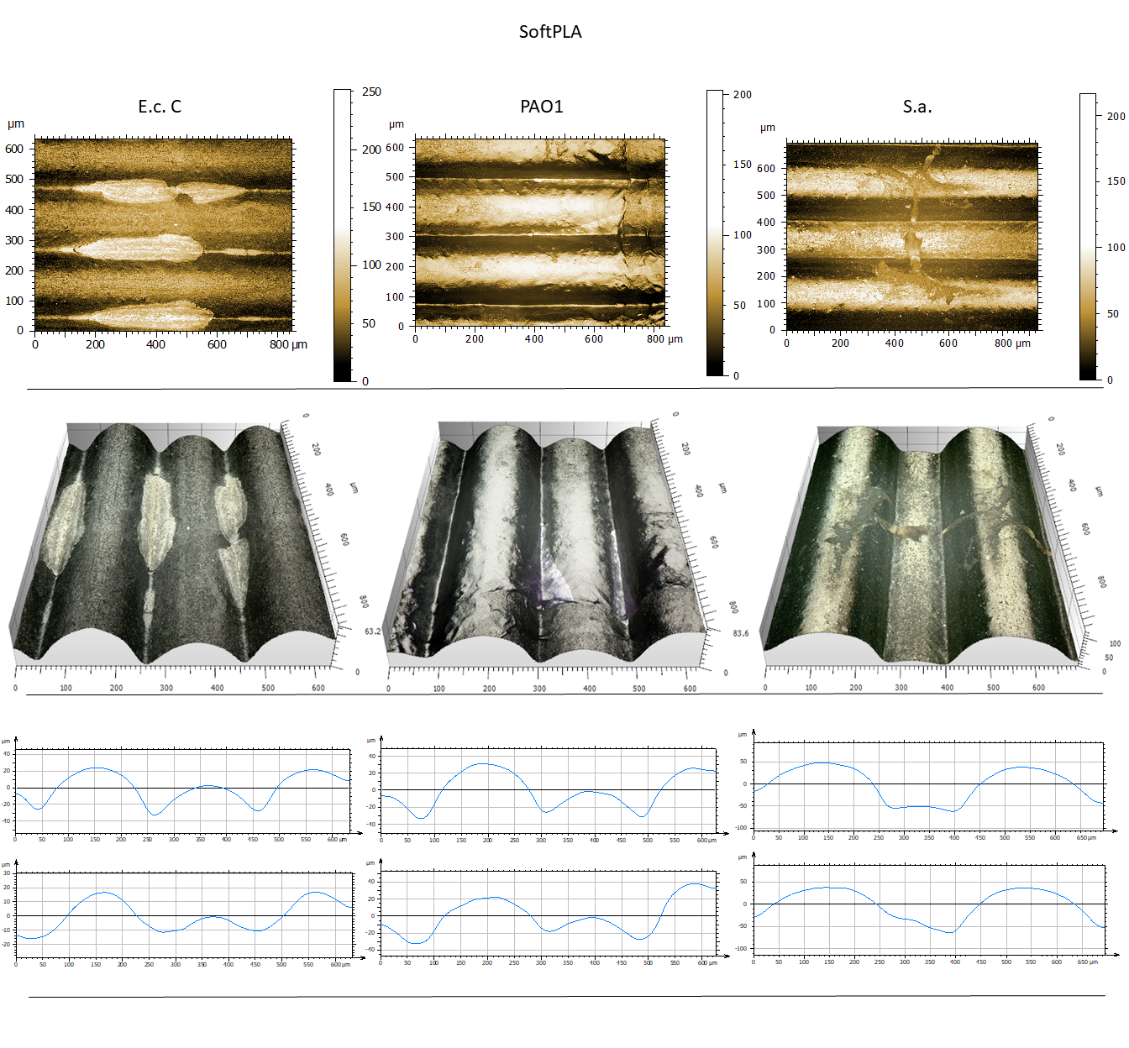


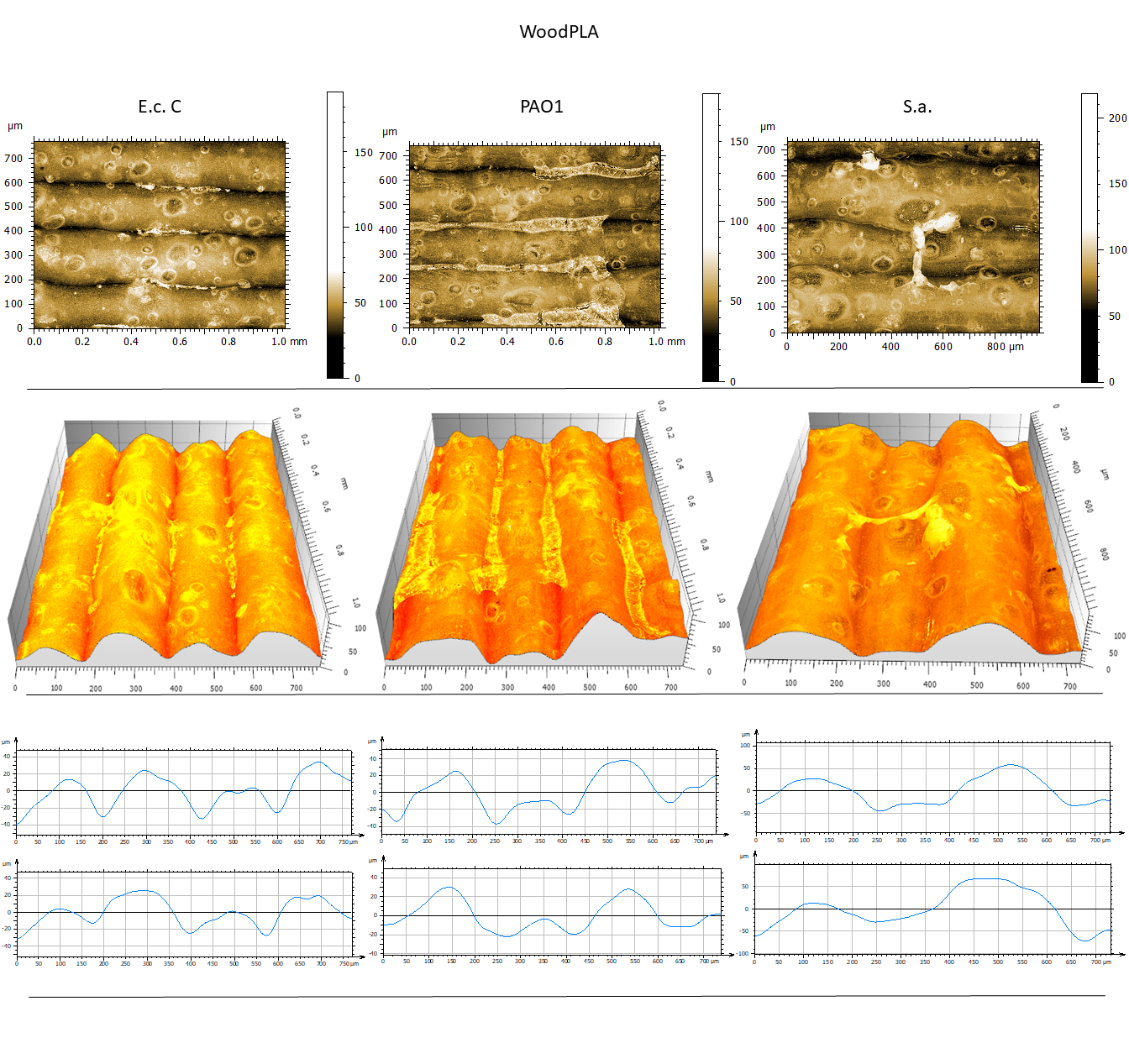


**Figure S6. Pictures of representative biofilms formed by *E. coli* C, *P. aeruginosa* PA01, and *S. aureus* ATCC25823 on 3D printed materials.** Upper panel: 2D pictures, Center panel: 3D projections, Bottom panel: projections of profiles without (upper) and with biofilms (lower) generated by MountainView® software.


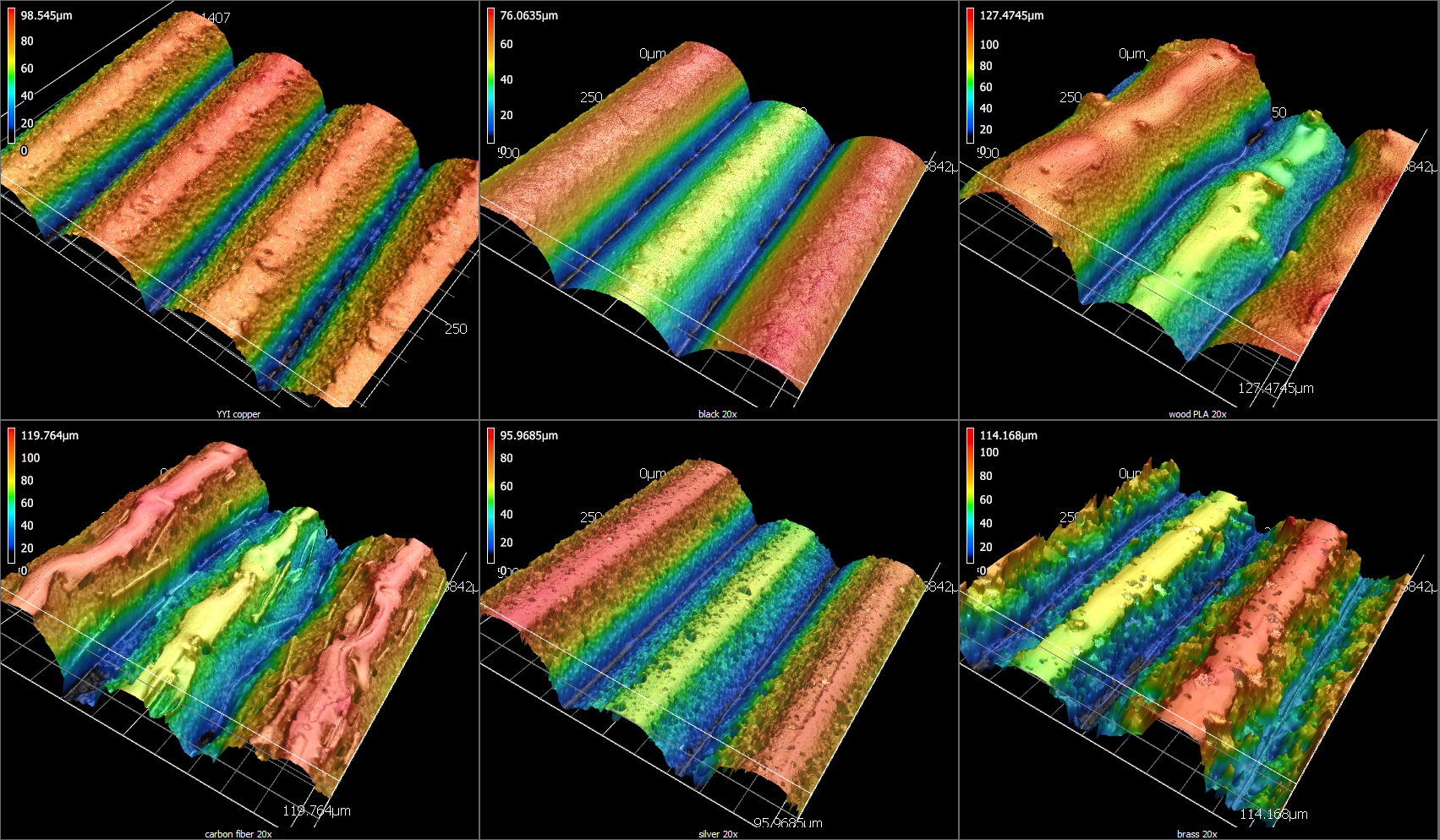


**Figure S**7. **3D projections of selected printouts obtained with Keyence VHX-7000 Laser Scanning Confocal Microscope.** From left top: CU, SoftPLA, WD, CF, BRS, BRZ.


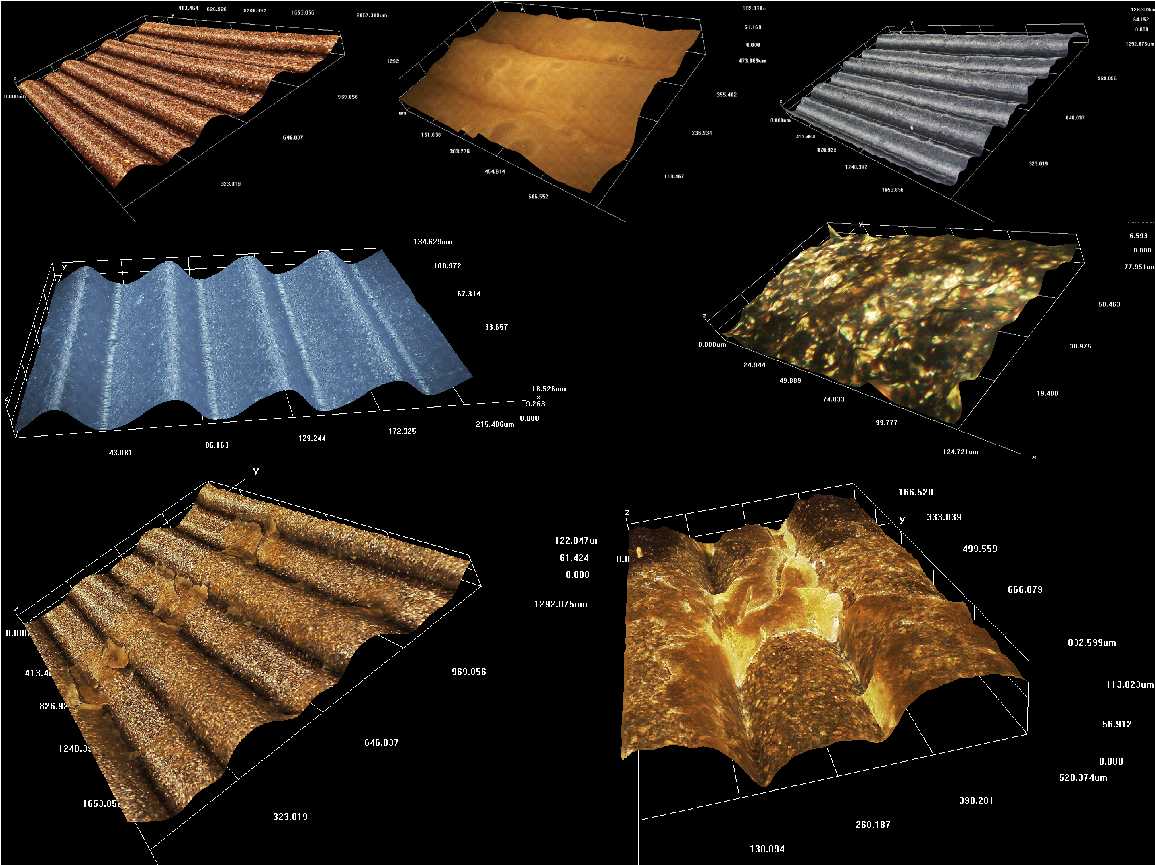


**Figure S8.** **3D projections of selected printouts obtained with Hirox KH8700 microscope.** From left top: CU, WD, SoftPLA, PLAS, BRS. Bottom: *P. aeruginosa* and *S. aureus* biofilms on CU (left and right, respectively)
